# Effect of Thermal Processing Parameters on the Tensile Properties of FDM-Printed ABS Components

**DOI:** 10.3390/polym18202491

**Published:** 2026-10-12

**Authors:** Saad M. S. Mukras, Hanafy M. Omar, Saleh Alzughaibi

**Affiliations:** Department of Mechanical Engineering, College of Engineering, Qassim University, Buraidah 51452, Saudi Arabia; hanafy@qu.edu.sa (H.M.O.); s.alzughaibi@qu.edu.sa (S.A.)

**Keywords:** fused deposition modeling, ABS, thermal processing parameters, response surface methodology, tensile properties, process optimization

## Abstract

Fused Deposition Modeling (FDM) offers manufacturing flexibility for acrylonitrile butadiene styrene (ABS), but mechanical performance is sensitive to thermal history during printing. This study examines the effects of printing temperature (PT), chamber temperature (CT), and cooling fan speed (CFS) on the tensile properties of FDM-printed ABS. A face-centered central composite design (75 specimens, 15 conditions, five replicates) was used to fit second-order models for ultimate tensile strength (UTS), tensile toughness (T, calculated as the area under the stress–strain curve up to fracture), and break strain ($\varepsilon_{B}$). PT was the dominant factor for all three responses (15–29% of total variance), with lower PT yielding higher strength, toughness, and ductility. A significant chamber-temperature × cooling-fan-speed interaction was identified for UTS (F=9.13, p=0.004), requiring joint optimization for strength. CT had no statistically significant effect on toughness or break strain, either alone or in interaction, within the tested range. Single-response optimization identified PT = 220 °C, CT = 42 °C, CFS = 50% as maximizing UTS (29.6 MPa); the point estimates for toughness and break strain were nominally higher at CT = 58 °C, but a formal contrast between CT = 42 and 58 °C showed 95% confidence intervals that included zero for both responses, so this difference cannot be resolved from noise. PT = 220 °C and CFS = 50% are robust recommendations across all models and weightings tested, while the optimal CT for toughness and break strain remains unresolved within this design. These findings provide a statistically grounded framework for selecting thermal process parameters for FDM-printed ABS components.

## 1. Introduction

Material extrusion additive manufacturing (MEX), commonly referred to as Fused Deposition Modeling (FDM) or Fused Filament Fabrication (FFF), has emerged as one of the most widely adopted additive manufacturing technologies due to its low cost, ease of operation, material versatility, and ability to fabricate complex geometries directly from digital models. The technology has expanded rapidly beyond prototyping into mainstream industrial applications, establishing its role in aerospace, automotive, medical, and consumer product sectors. Despite its increasing industrial adoption, the mechanical performance of FDM-manufactured components remains a significant challenge due to process-induced anisotropy, interlayer defects, porosity, and residual stresses generated during layer-by-layer fabrication [1,2,3].

Among the thermoplastic materials available for FDM processing, acrylonitrile butadiene styrene (ABS) remains one of the most widely used engineering polymers because of its excellent balance of strength, toughness, impact resistance, thermal stability, and cost-effectiveness. ABS is commonly employed in automotive interiors, consumer products, functional prototypes, and lightweight structural components. Nevertheless, ABS is prone to processing difficulties; its relatively high coefficient of thermal expansion means that deposited layers cool and contract at different rates, generating internal thermal stresses that can lead to warping and delamination if not managed through careful process control. Consequently, understanding the relationship between thermal processing conditions and the resulting mechanical properties of FDM-printed ABS components represents a critical research objective [4,5].

The mechanical behavior of FDM-manufactured polymers is strongly governed by the quality of interlayer bonding formed during deposition. The bonding quality among polymer filaments in the FDM process determines the integrity and mechanical properties of resultant parts; the development of these bonds depends on molecular diffusion and chain entanglement between adjacent filaments while the material remains above its glass transition temperature. Interlayer bonding strength is governed by coalescence processes, resulting from phenomena such as surface wetting, neck growth, and interfacial polymer chain diffusion and entanglements [6,7]. Consequently, process parameters that influence thermal history directly affect the toughness, break strain, ultimate tensile strength, and failure mechanisms of printed components. Thermal management is becoming a primary concern in FDM optimization. While earlier studies focused on geometric parameters such as raster orientation, infill density, layer thickness, and build orientation, other investigations have emphasized the role of thermal history in controlling interlayer bonding quality and mechanical performance [6].

### 1.1. Effect of Printing Temperature

Printing temperature is widely recognized as one of the most influential processing parameters affecting the mechanical behavior of FDM-fabricated components. The extrusion temperature determines polymer melt viscosity, filament flow characteristics, molecular mobility, and the extent of interlayer diffusion during deposition. Increasing nozzle temperature generally enhances polymer chain mobility and prolongs the period during which deposited filaments remain in a softened state, thereby promoting stronger interlayer welding and improved load transfer across layer interfaces [6,8]. Abbott et al. [9] assessed bond strength as a function of print parameters including extruder temperature, print speed, and layer height using infrared thermography to track the thermal history at interlayer bond lines; the study demonstrated that deposition temperature directly governs the extent of polymer chain interpenetration and interface contact length, with a plateau in tensile strength of 22 MPa observed for a normalized contact length greater than 0.6. More recently, Ulkir et al. [10] investigated the effect of nozzle temperature on the tensile strength, hardness, and dimensional properties of FDM-ABS specimens, varying the nozzle temperature from 220 °C to 270 °C in 10 °C increments across thirty-six ISO 527 Type 1A tensile specimens. One-way ANOVA confirmed that nozzle temperature is a statistically significant factor affecting all measured mechanical and dimensional outputs. Tensile strength decreased by 41.52% and Shore D hardness decreased by 14.59% as the nozzle temperature increased from 220 °C to 270 °C, with the highest tensile strength of 24.59 MPa recorded at the lowest tested temperature of 220 °C. Scanning electron microscopy (SEM) analysis of the fracture surfaces revealed that elevated temperatures progressively increased the void content within the printed layers, reducing both specimen mass (by 55.95%) and mechanical integrity. These findings underscore the critical importance of nozzle temperature selection in FDM processing of ABS and motivate systematic investigation of its interaction with other process parameters.

### 1.2. Effect of Chamber Temperature

The thermal environment surrounding the printed component plays a critical role in determining cooling behavior and residual stress development. Chamber temperature influences the temperature gradient between the deposited filament and the surrounding air, thereby affecting cooling rates, thermal contraction, and stress accumulation during solidification. The layer-by-layer deposition characteristic of FDM produces rapid heating and cooling cycles that introduce residual stress during the build-up process which may cause warping, delayering, and distortion. High-level printing systems use a heated chamber to mitigate these defects. Elevated chamber temperatures reduce thermal gradients, prolong the interdiffusion process between adjacent layers, and decrease shrinkage-induced stresses, collectively contributing to improved interlayer bonding and dimensional stability. Simulation studies have shown that increasing ambient temperature from 50 °C to higher values results in measurable decreases in residual stress in ABS, a direct consequence of the reduced thermal gradients experienced by the material during fabrication [11].

Experimentally, Cuc et al. [12] investigated 3D printing in various environmental temperatures using FDM and demonstrated the influence of printing environment temperature on the mechanical properties of ABS, reporting improvements in tensile performance at elevated ambient conditions. Despite this growing evidence, research on the influence of chamber temperature on the mechanical properties of FDM-printed components remains comparatively limited relative to other process parameters.

### 1.3. Thermal History Control Through Cooling Conditions

In addition to printing and chamber temperatures, cooling conditions represent a third major factor controlling the thermal history of deposited filaments. Cooling fan speed directly affects convective heat transfer from the deposited bead and determines the rate at which material loses thermal energy after extrusion. While rapid cooling may improve dimensional accuracy and surface finish, excessive heat removal shortens the time available for molecular diffusion and interfacial healing, potentially weakening interlayer bonds. For ABS in particular, poor interlayer bonding is exacerbated by high fan speeds, and common practice recommends that the cooling fan be off or set to a low speed when printing ABS to preserve interlayer adhesion quality. At the interface between successive layers, elevated temperatures increase molecular motion, enabling polymer chains from each layer to interpenetrate. This interdiffusion produces entanglements that significantly enhance bond strength, a process directly curtailed by aggressive cooling. Despite its practical importance, cooling fan speed remains among the least systematically studied thermal parameters in FDM, particularly for ABS materials printed in heated build environments [12,13].

### 1.4. Research Gap and Objectives

Although considerable research has been devoted to understanding the influence of individual FDM process parameters on mechanical performance, most studies have investigated thermal variables independently, and existing investigations frequently focus on nozzle temperature optimization while treating chamber temperature and cooling conditions as secondary parameters. The mechanical properties of 3D-printed products depend on many factors, of which complete control over temperature within the building chamber is among the most important; yet studies on the combined influence of these thermal-control variables remain minimal. More importantly, printing temperature, chamber temperature, and cooling fan speed collectively define the thermal history experienced by the deposited material and therefore govern polymer diffusion, interlayer welding, residual stress development, and final mechanical performance. Furthermore, previous studies have predominantly focused on ultimate tensile strength, while comparatively limited attention has been given to the simultaneous response of toughness, break strain, and ultimate tensile strength across a designed parameter space.

Therefore, the objective of the present study is to investigate the effects of three key thermal processing parameters: printing temperature, chamber temperature, and cooling fan speed on the tensile properties of FDM-printed ABS specimens. Tensile testing was conducted to evaluate the ultimate tensile strength, toughness, and break strain of the printed samples. By establishing relationships between these thermal processing conditions and the resulting mechanical performance, this study seeks to advance the understanding of thermal-history effects in FDM-manufactured ABS and contribute to the optimization of process parameters for structural engineering applications.

## 2. Materials and Methods

### 2.1. Material

The material used in this study was an acrylonitrile butadiene styrene (ABS) thermoplastic filament supplied by eSUN (Shenzhen, China), with a nominal diameter of 1.75 mm. ABS was selected owing to its well-established suitability for Fused Deposition Modeling (FDM) processing and its widespread use in engineering, automotive, and consumer product applications requiring a combination of mechanical strength, thermal resistance, and toughness.

The bulk material properties of the eSUN ABS filament, as reported in the manufacturer’s Technical Data Sheet and measured on injection-molded specimens, are presented in Table 1. It should be noted that these values represent the bulk polymer properties and are expected to differ from those of FDM-fabricated specimens, which are subject to process-induced anisotropy and interlayer bonding effects.

The filament spool was stored in a sealed, low-humidity environment prior to printing to minimize moisture absorption, which is known to adversely affect surface quality and interlayer bonding in FDM-processed ABS. The manufacturer’s recommended printing conditions, as specified in the TDS and used as the basis for selecting experimental parameter ranges in this study, are summarized in Table 2 (included on the product packaging).

### 2.2. Specimen Design and Fabrication

#### 2.2.1. Specimen Geometry and Testing Standard

**Figure 1:**
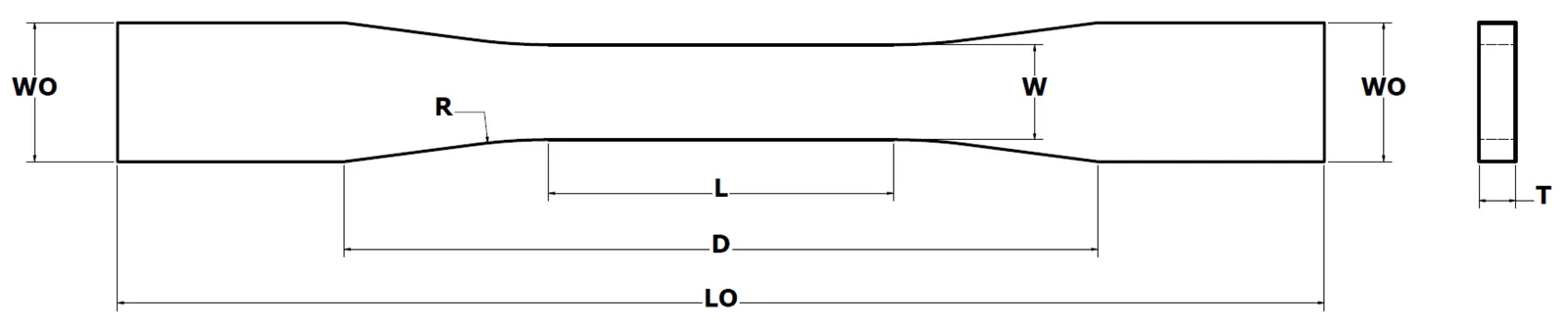
Dog-bone geometry for the ASTM D638 Type I tensile specimen.

Tensile specimens were designed and fabricated in accordance with ASTM D638 Standard Test Method for Tensile Properties of Plastics [15] using the Type I dog-bone geometry (see Figure 1). ASTM D638 Type I is the most widely adopted specimen configuration for rigid and semi-rigid thermoplastics and has been extensively employed in previous FDM tensile characterization studies for ABS [3,16], allowing direct comparison of the results with the published literature. The principal dimensions of the ASTM D638 Type I specimen are provided in Table 3.

#### 2.2.2. 3D Printer and Equipment

**Figure 2:**
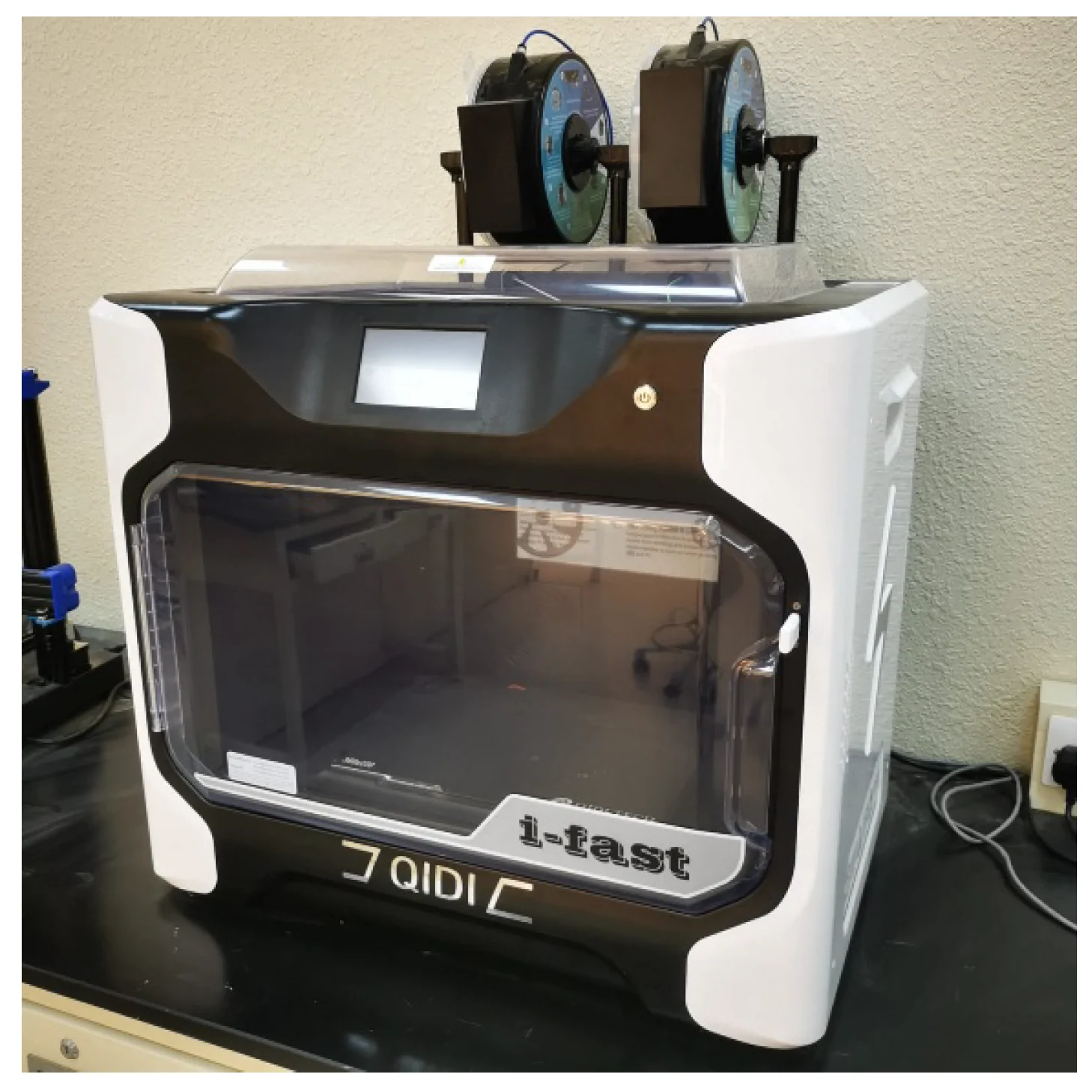
QIDI i-Fast FDM 3D printer used in the study.

All specimens were fabricated using a QIDI i-Fast Fused Deposition Modeling (FDM) 3D printer (QIDI Technology Co., Ltd., Wenzhou, China), depicted in Figure 2. The i-Fast is a fully enclosed, industrial-grade dual-extruder system equipped with an actively heated build chamber capable of reaching temperatures up to 60 °C, dual linear-guide-rail Z-axis motion for dimensional stability, and a 330 × 250 × 320 mm build volume. The enclosed chamber design of the i-Fast makes it particularly well-suited to the fabrication of ABS components, which are susceptible to warpage and interlayer delamination under uncontrolled thermal environments.

A standard 0.4 mm diameter brass nozzle was used throughout all experimental trials to ensure consistent extrusion geometry. Specimen tool paths and G-code were generated using QIDI Print slicing software (QIDI Print Slicer, version V6.2.0). The principal technical specifications of the printer are listed in Table 4.

#### 2.2.3. Fixed Printing Parameters

To isolate the effects of the three independent thermal process variables (printing temperature, chamber temperature, and cooling fan speed) all other printing parameters were held constant across the entire experimental program. The fixed parameters and their selected values are listed in Table 5.

A 100% infill density was specified to minimize the confounding influence of internal void fraction on measured tensile properties, ensuring that differences in mechanical performance can be attributed solely to variations in the thermal process parameters under investigation. Even at a nominal 100% infill, the circular/oval cross-section of the extruded bead leaves small interstitial gaps between adjacent rasters, so this setting reduces, but does not remove, internal voids. The rectilinear zigzag infill pattern at ±45° to the specimen longitudinal axis was selected as a commonly adopted raster configuration for FDM tensile specimens, providing a balance between longitudinal and transverse reinforcement. The build platform temperature was maintained at 100 °C throughout all trials to ensure consistent first-layer adhesion, within the manufacturer’s recommended range of 95–110 °C for ABS.

### 2.3. Experimental Design

#### 2.3.1. Design Strategy and Rationale

A face-centered central composite design (FC-CCD) was employed to investigate the effects of the three independent thermal process variables (printing temperature (PT), chamber temperature (CT), and cooling fan speed (CFS)) on the tensile properties of FDM-printed ABS specimens.

The FC-CCD was selected over a full three-level factorial design ($3^{3}=27$ conditions) because it provides equivalent capability for fitting a full quadratic polynomial model, capturing linear, quadratic, and two-way interaction effects with a substantially reduced number of experimental conditions. For three factors, the FC-CCD requires only 15 unique conditions (8 factorial points, 6 axial face-center points and 1 center point) compared to 27 for a full factorial, representing a 44% reduction in experimental burden without loss of model resolution.

In the face-centered variant, the axial points are placed on the faces of the factorial cube rather than outside it, ensuring all experimental points remain within the physically feasible parameter space. This characteristic is particularly important for the present study, where the factor ranges were set based on practical limits of the QIDI i-Fast printer and the thermal sensitivity of ABS, and where extrapolation beyond these bounds would be physically unreasonable.

The FC-CCD yields a three-level design, where each factor takes a low (−1), center (0), and high (+1) coded value, as detailed in Table 6. The bounds of the three thermal process variables used in the study are also shown in Table 6.

Printing temperature was varied between 220 °C and 260 °C, centered at 240 °C. This range spans the lower and upper bounds of the manufacturer’s recommended extrusion window for eSUN ABS (as stated in the product packaging) and is similar to values reported in published ABS tensile studies [10]. Chamber temperature was varied between 42 °C and 58 °C, centered at 50 °C.

A face-centered CCD is not rotatable, so the prediction variance of the fitted surface is not constant at equal distances from the design center, and the pure-quadratic coefficients are estimated less precisely than they would be in a rotatable CCD. This design was nonetheless chosen because a rotatable design requires axial points outside the factorial cube, which the physical limits of the printer do not allow (for example, the chamber temperature cannot exceed the equipment’s 60 °C rating). The five replicates collected at every condition support the pure-error estimate, and the main conclusions of this study rely primarily on the linear and two-factor interaction terms, which the face-centered design estimates well.

#### 2.3.2. Experimental Run Matrix

The complete experimental program comprised 15 unique process conditions, as listed in Table 7. These consist of 8 factorial corner points (S01–S08), 6 axial face-center points (S09–S14), and 1 center point (S15). The center point serves to provide an estimate of pure experimental error and to detect curvature in the response surface, enabling the adequacy of a quadratic model to be evaluated.

Five replicate specimens were fabricated and tested at each of the 15 conditions, yielding a total of 75 tensile specimens. All 75 specimens were printed in a fully randomized order, determined prior to fabrication using a random number generator, to minimize the effects of systematic environmental drift and printer warm-up effects on the measured responses.

The chamber temperature was set on the printer’s controller and was verified using an HT-9815 4-channel digital thermometer with a K-type thermocouple, used as supplied without independent calibration and placed adjacent to the extruder. The chamber and bed were preheated for more than 30 min before each print, and printing started once the target temperature was confirmed. Continuous temperature logging was not performed, and the spatial distribution of temperature within the chamber volume was not mapped; the reported chamber temperature therefore refers specifically to the value measured near the extruder. Specimens were printed in randomized order, so any systematic gradient or drift would not be confounded with a single factor level.

In this study, non-reinforced ABS was used (nozzle specifications are given in Table 4), and the total filament consumed over the 75 specimens was slightly under 1000 g. Since nozzle wear is mainly associated with abrasive filaments, such as carbon-fiber, glass-fiber or metal-filled materials, significant erosion of the 0.4 mm brass nozzle orifice was not expected at this throughput with a non-abrasive material. The specimens were printed in randomized order, so any residual effect of nozzle wear would not be systematically associated with a particular parameter level.

After printing, the specimens were stored at ambient laboratory conditions (approximately 23 °C, 35% RH) in sealed containers and were tested more than 5 days after printing, in randomized order, over several testing sessions spanning 4 days. No standardized conditioning (e.g., according to ASTM D618) or drying was applied to the specimens, and the filament was used as received from the manufacturer’s sealed packaging. The absolute property values reported may therefore differ from those obtained with standard conditioning, and the results should be considered specific to the ABS filament, printer and conditions used in this study rather than representative of ABS in general. Because all specimens were treated identically and printed in randomized order, the comparison between parameter levels, which is the focus of this study, is not affected by this.

### 2.4. Tensile Testing

**Figure 3:**
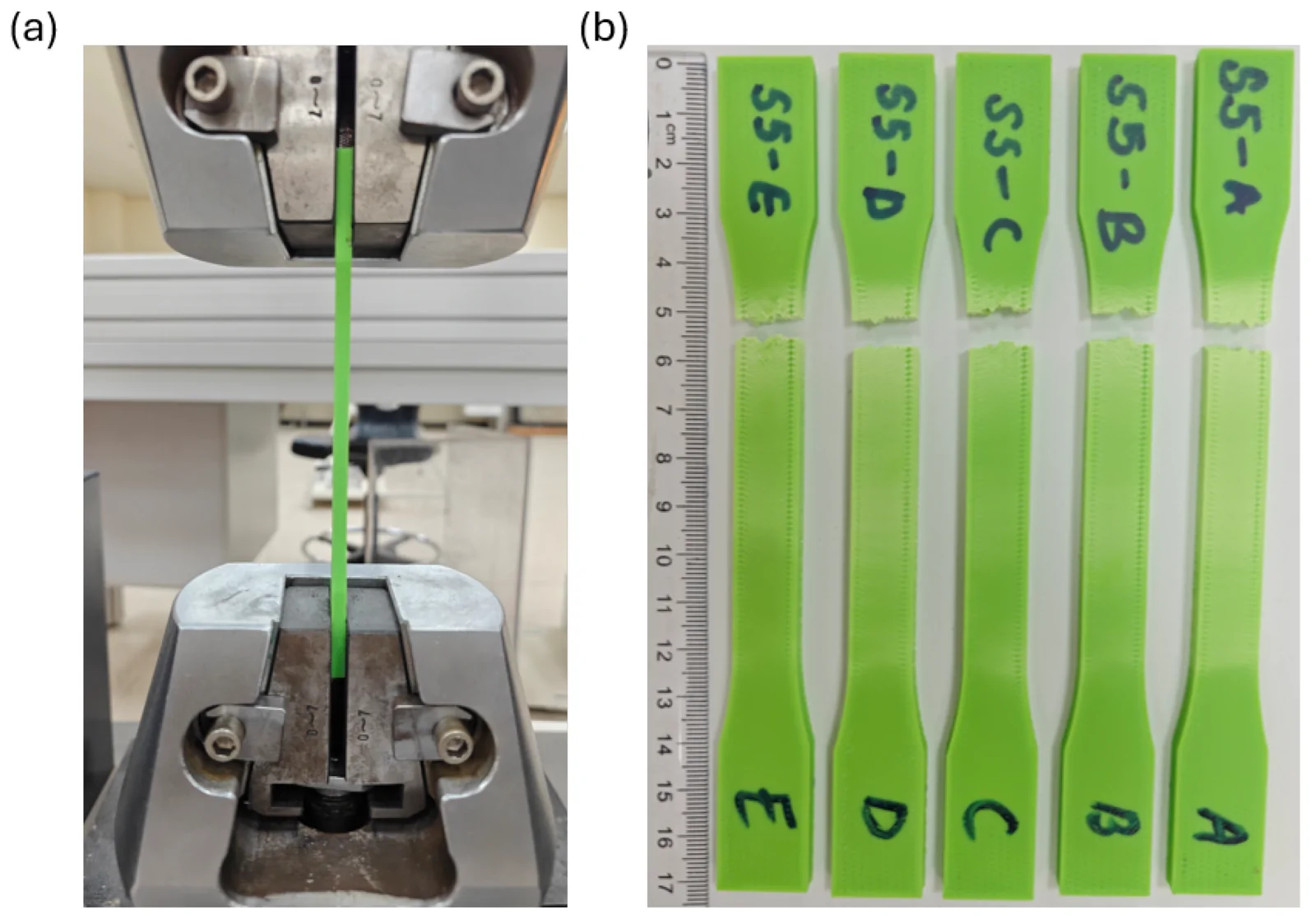
Tensile testing setup and representative specimens. (**a**) Specimen under uniaxial tensile testing. (**b**) Five replicates after failure (A–E).

Uniaxial tensile tests were conducted based on ASTM D638 to evaluate the mechanical properties of 3D-printed ABS specimens fabricated under different printing conditions. The tests were performed at an ambient temperature of approximately 23 °C using a TESTCOM 100 universal testing machine (IBERTEST, Madrid, Spain) equipped with a 100 kN load cell. The specimens were mounted using mechanical wedge grips and tested under displacement control at a crosshead speed of 1 mm/min, as shown in Figure 3a. This speed was selected to approximate quasi-static loading conditions and reduce the influence of loading-rate effects on the comparison between printing conditions. Force and crosshead displacement were recorded continuously until failure. The gauge length, width, and thickness of the specimens were 57 mm, 13 mm, and 4.8 mm, respectively. Five replicate specimens were tested for each condition, as represented in Figure 3b.

Engineering stress was calculated using the measured force and the original cross-sectional area, while engineering strain was obtained from the crosshead displacement normalized by the initial gauge length. The stress–strain curves were corrected for the initial toe region before calculating strain-dependent properties. Ultimate tensile strength was defined as the maximum engineering stress. Toughness was calculated as the area under the corrected engineering stress–strain curve up to the point of fracture. Results are presented as mean ± standard deviation for each condition.

### 2.5. Statistical Analysis

For each response *Y*, a full second-order polynomial in the three coded factors $x_{1}=\left(\mathrm{PT}-240\right)/20$, $x_{2}=\left(\mathrm{CT}-50\right)/8$ and $x_{3}=\left(\mathrm{CFS}-25\right)/25$ was fitted by ordinary least-squares (OLS) regression, Equation (1):

(1) $$Y=\beta_{0}+\beta_{1}x_{1}+\beta_{2}x_{2}+\beta_{3}x_{3}+\beta_{12}x_{1}x_{2}+\beta_{13}x_{1}x_{3}+\beta_{23}x_{2}x_{3}+\beta_{11}x_{1}^{2}+\beta_{22}x_{2}^{2}+\beta_{33}x_{3}^{2}+\varepsilon$$

where $\beta_{0}$ is the intercept; $\beta_{1}$, $\beta_{2}$, $\beta_{3}$ are the linear coefficients for $x_{1}$, $x_{2}$ and $x_{3}$ respectively; $\beta_{12}$, $\beta_{13}$, $\beta_{23}$ are the two-factor interaction coefficients; $\beta_{11}$, $\beta_{22}$, $\beta_{33}$ are the quadratic coefficients; and $\varepsilon ∼N(0,\sigma^{2})$ is the random error term. Coefficient significance was assessed by two-sided *t*-tests on each coefficient; term significance was confirmed by a Type-II analysis of variance (ANOVA). Model adequacy was evaluated through R^2^, adjusted R^2^, the global F-statistic, and a formal lack-of-fit test that partitions the residual sum of squares into a pure-error component (estimated independently of the model from the within-condition replicate variance) and a lack-of-fit component. The residuals were inspected for normality (Shapiro–Wilk test), homoscedasticity (Breusch–Pagan test) and influential observations (Cook’s distance with the 4/*n* threshold). Practical importance of every model term was quantified through three complementary effect-size measures from the Type-II ANOVA: percent of total sum of squares (%SS), partial $\eta^{2}$ and bias-corrected $\omega^{2}$. Single-response optima were located by bounded multi-start L-BFGS-B maximization of each fitted polynomial over the coded design region.

Five starting points, distributed across the coded cube, were used to guard against convergence to a local optimum. Multi-response (joint) optima were obtained with the Derringer–Suich desirability function, with the overall desirability surface additionally evaluated on a dense 21×21×21 grid over the coded factor cube as a check on the gradient-based search. All regression fitting used ordinary least squares, term significance was assessed with the Type-II ANOVA, and the lack-of-fit test, residual normality (Shapiro–Wilk), homoscedasticity (Breusch–Pagan), Cook’s distance, HC3-robust standard errors and the PRESS statistic were computed with the corresponding statsmodels and scipy routines.

All statistical analyses were performed in Python 3 (3.12) using the statsmodels (0.14.4) and scipy (1.14.1) libraries, with α=0.05 throughout.

## 3. Results

### 3.1. Mechanical-Property Variability and Repeatability

**Figure 4:**
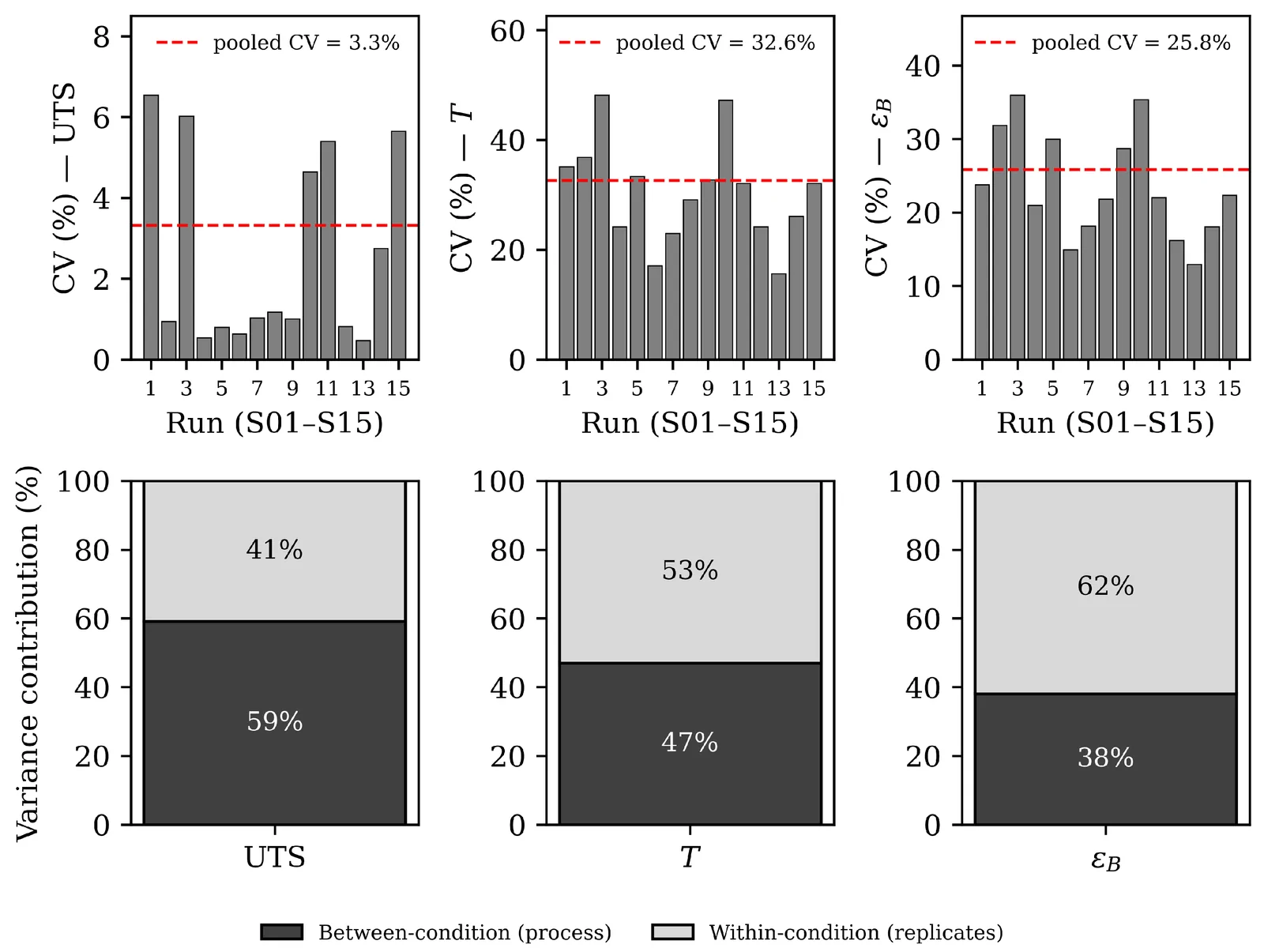
Within-condition CV per run ((**top**); dashed line = pooled CV) and variance decomposition into between- and within-condition components (**bottom**) for the three responses.

Before interpreting the factor effects, the repeatability of the printing-plus-testing pipeline was characterized, since the magnitude of replicate scatter sets the floor of what any subsequent regression model can hope to explain. For every response the pooled within-condition standard deviation $\sigma_{pooled}$ was computed by pooling the variance across the 15 conditions, and the corresponding coefficient of variation $CV=\sigma_{pooled}/\bar{Y}$ was evaluated. A variance partition was then performed to assign the total response variance to a between-condition (process-driven) component and a within-condition (replicate-driven) component. Levene’s test (median-centered, robust variant) was used to test the homogeneity of the within-condition variance across the design region. The numerical results are summarized in Table 8, and the corresponding visualizations appear in Figure 4.

A sensitivity power analysis was carried out using the residual standard deviation of the full quadratic models fitted to all 75 runs. For a low-to-high change in a single factor to be detected at 80% power (α=0.05), the required effect size is approximately 3.1% of the mean for UTS, but about 28% of the mean for toughness and 22% for break strain. Considered instead as a two-sample comparison between two single conditions with five specimens each, as used in Section 3.8 for the CT contrast, the smallest reliably detectable difference is approximately 7% (UTS), 66% (toughness) and 52% (break strain) of the response mean. Five replicates per condition are therefore adequate to resolve the effects reported for UTS, but are under-powered for smaller effects on toughness and break strain; the absence of a statistically significant CT effect on these two responses should accordingly be read as “no effect was detected at this replication level”, not as evidence that no effect exists. More replicates per condition are recommended in future work to resolve smaller effects on toughness and break strain.

For UTS the pooled coefficient of variation is low (CV ≈ 3.3%), indicating a highly repeatable manufacturing process for this strength metric. Toughness is markedly noisier (CV ≈ 32.6%), consistent with its sensitivity to the full post-yield stress–strain trajectory rather than a single linear region. The variance partition confirms that approximately 59% of the total variance in UTS is driven by the experimental factors, with 41% attributable to specimen-to-specimen scatter; for toughness the split is closer to 47/53%, reflecting a smaller process signal-to-noise ratio.

Break strain and toughness are both qualitatively different from UTS. Break strain’s pooled CV is approximately 26%, and toughness’s is even higher at approximately 33%—both almost an order of magnitude larger than UTS’s 3.3%—while only 38% (break strain) and 47% (toughness) of their total variance is attributable to between-condition effects. This behavior is characteristic of ductility- and fracture-related measurements in fused-filament fabrication, where final elongation and the energy absorbed to fracture are governed by local fracture initiation at sub-millimeter defects (voids, partial interlayer fusion, over- or under-extrusion) whose spatial distribution varies stochastically from print to print; toughness, being derived from the integrated stress–strain curve up to fracture, inherits this same sensitivity. The implication is that $\varepsilon_{B}$- and toughness-driven optimization both require more replication than UTS-driven optimization to reach the same statistical confidence, and that any reported optimum for either carries a wider confidence interval.

Levene’s test confirms the equal-variance assumption for toughness (p=0.84) and $\varepsilon_{B}$ (p=0.76), but rejects it for UTS (p=0.033), indicating mild heteroscedasticity in the strength data. The OLS coefficient estimates remain unbiased under heteroscedasticity for the present sample size; heteroscedasticity-consistent (HC3) standard errors used as a sensitivity check do not alter the set of statistically significant terms reported below.

### 3.2. Main Effects and Pareto Ranking of Factor Importance

**Figure 5:**
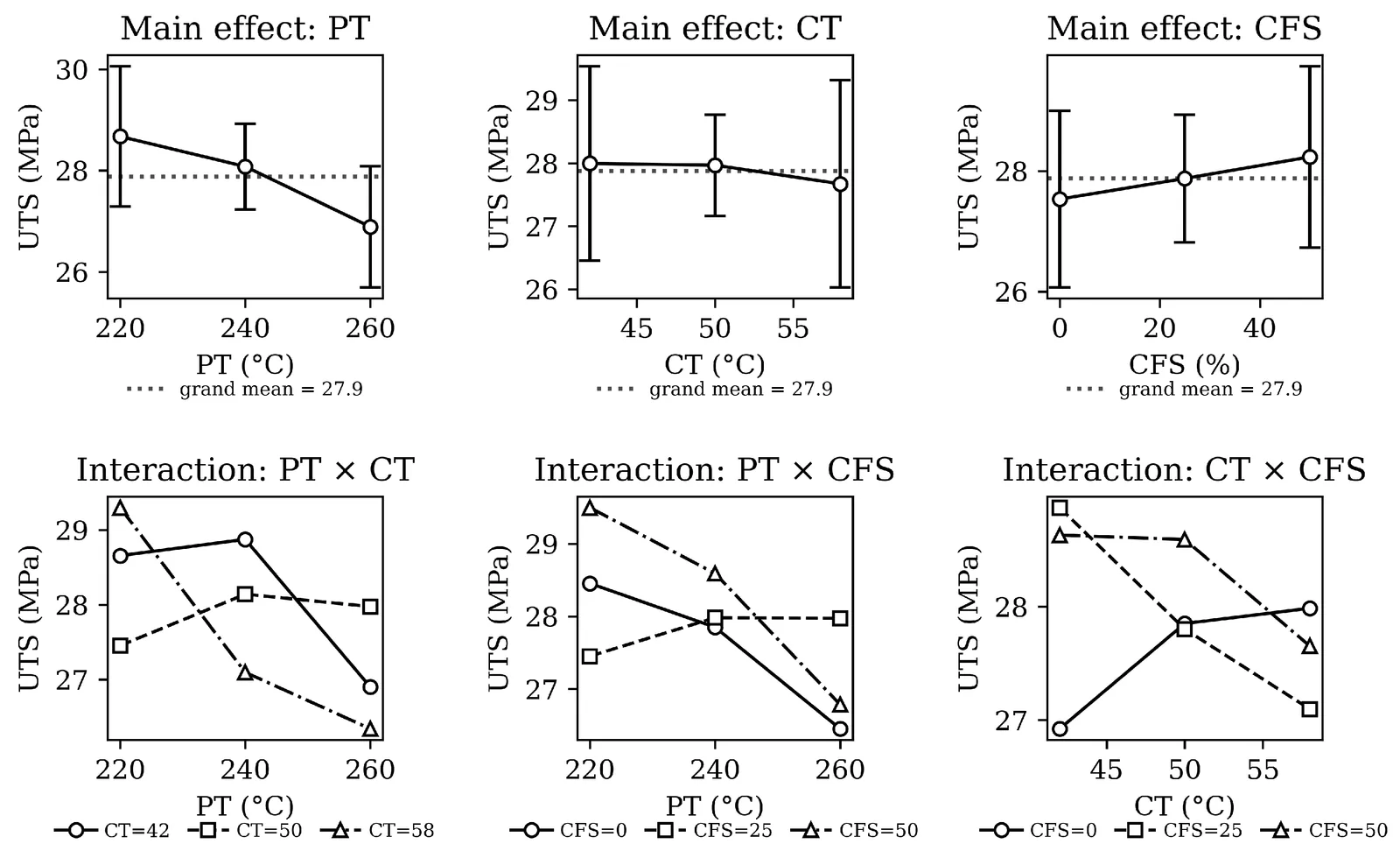
Main effects (**top**) and two-factor interactions (**bottom**) for UTS.

**Figure 6:**
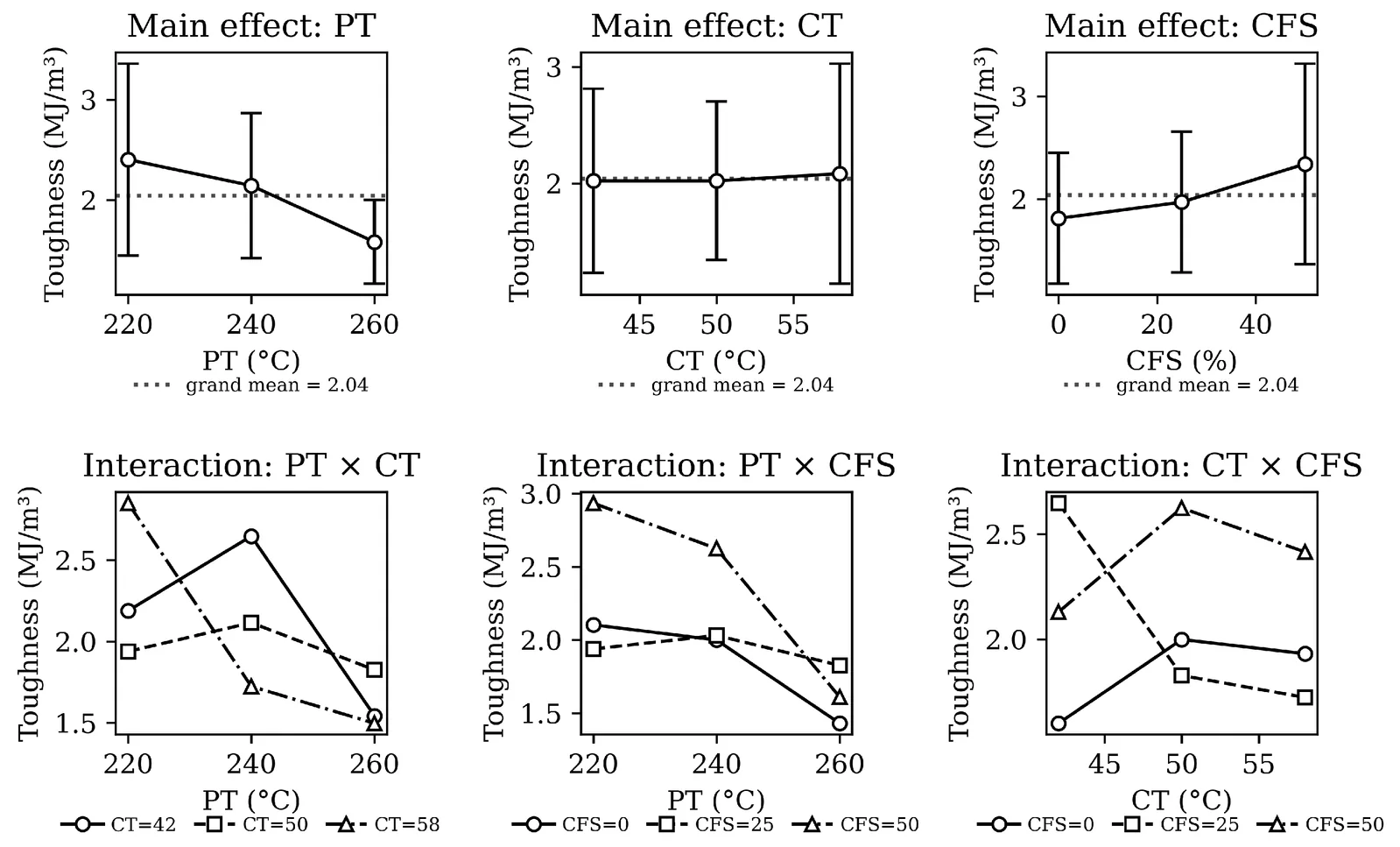
Main effects (**top**) and two-factor interactions (**bottom**) for toughness (*T*).

**Figure 7:**
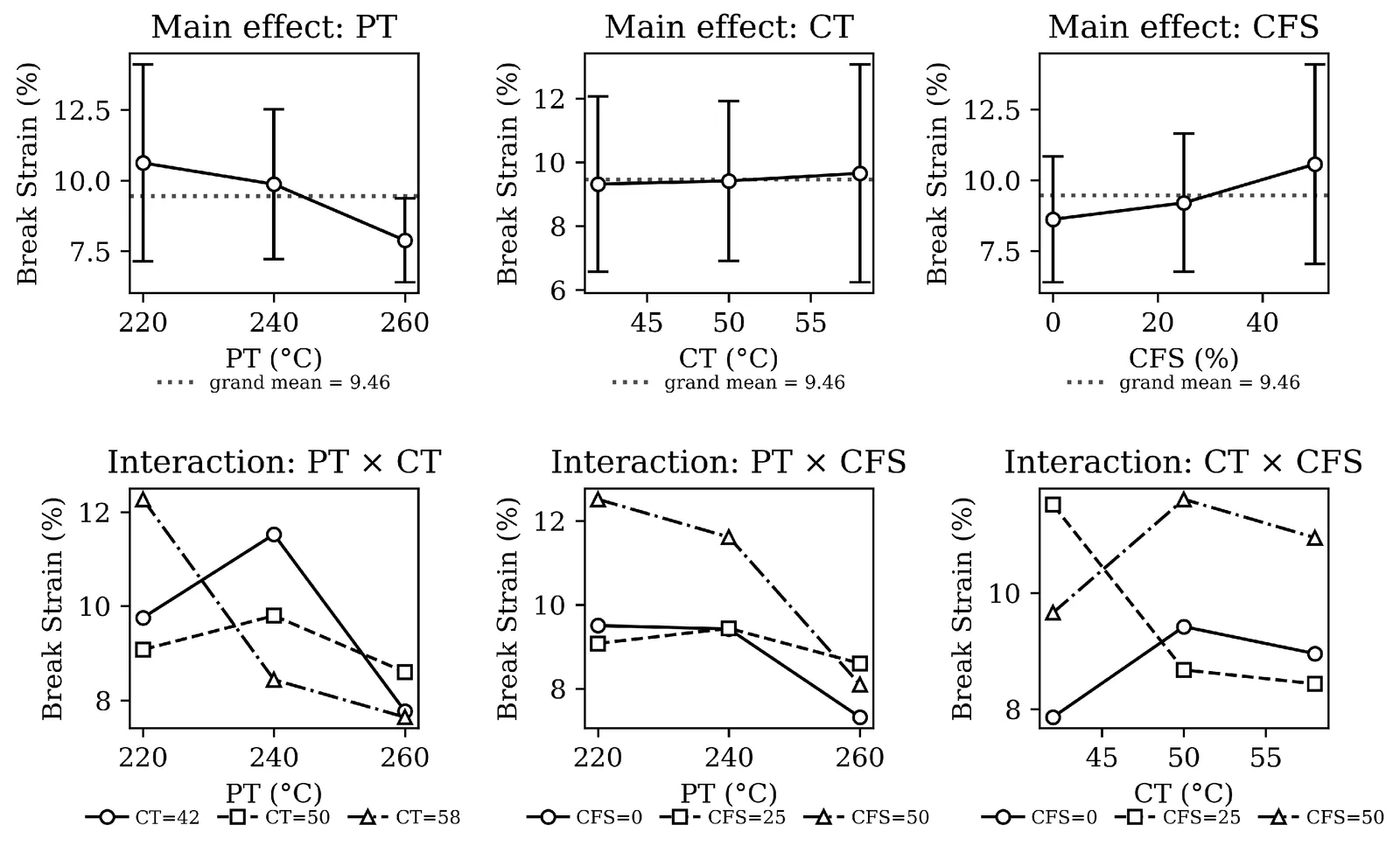
Main effects (**top**) and two-factor interactions (**bottom**) for break strain ($\varepsilon_{B}$).

Figure 5, Figure 6 and Figure 7 present, for each response, the main-effect plots (top row) showing the response averaged across replicates at each of the three levels of one factor, with error bars representing one within-level standard deviation. The dotted horizontal line marks the overall grand mean. The interaction panels in the bottom row are discussed in Section 3.3.

For UTS (Figure 5), the main effect of PT is the largest single trend in the data: the mean UTS falls from 28.7 MPa at PT = 220 °C to 26.9 MPa at PT = 260 °C—a 6% drop. CT exerts a much weaker main effect and CFS produces a mild positive trend. For toughness (Figure 6) PT is the dominant main effect (toughness decreases as PT rises) and CFS shows a mild positive trend, while CT alone is essentially flat and not statistically significant (F=0.10, p=0.758). For break strain (Figure 7) PT and CFS dominate: $\varepsilon_{B}$ falls by approximately 25% between PT = 220 °C and 260 °C and rises by approximately 20% from CFS = 0% to CFS = 50%; CT has a negligible main effect.

**Figure 8:**
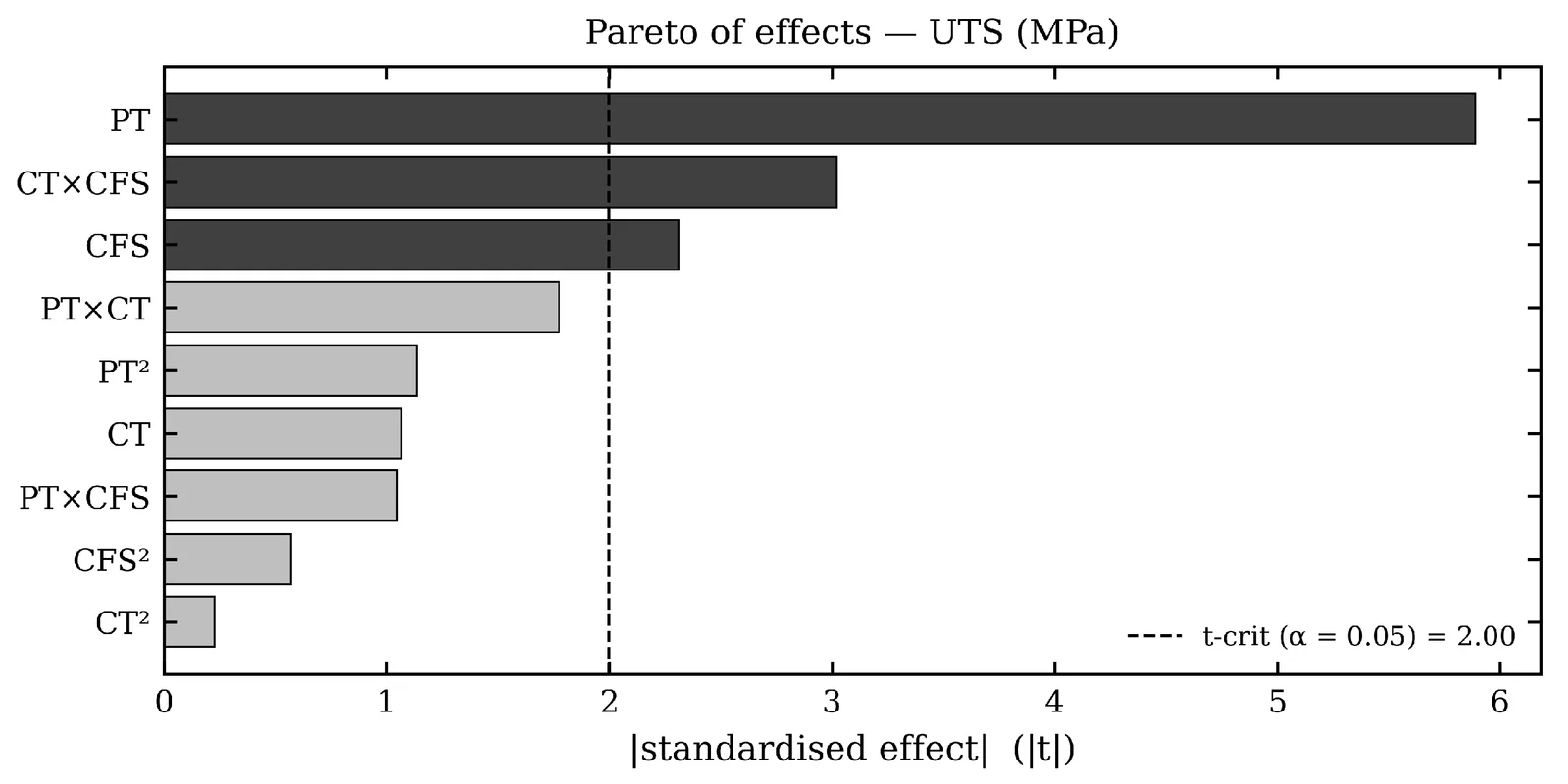
Pareto plot of standardized effects for UTS (dark = significant at α=0.05).

**Figure 9:**
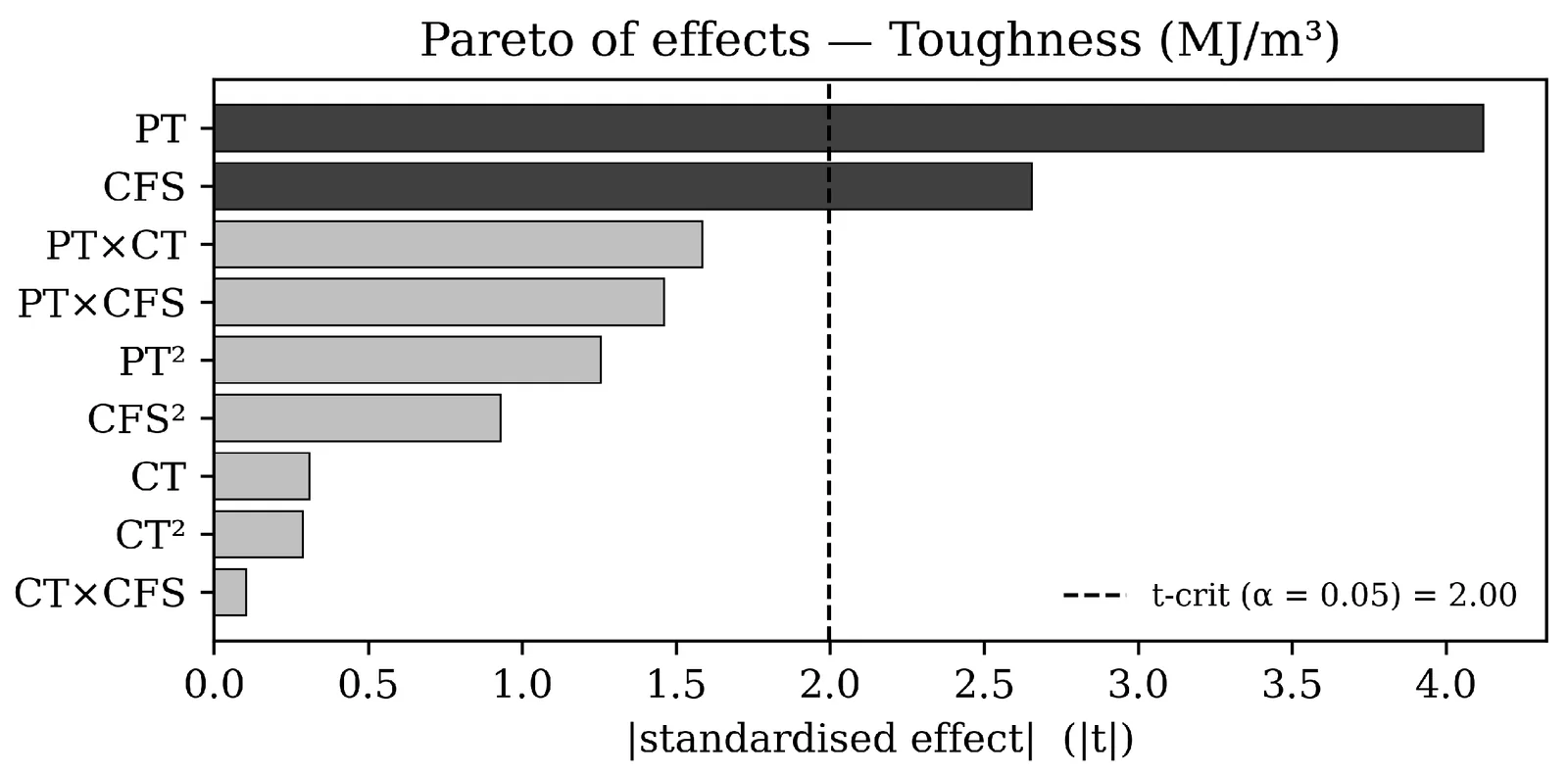
Pareto plot of standardized effects for toughness.

**Figure 10:**
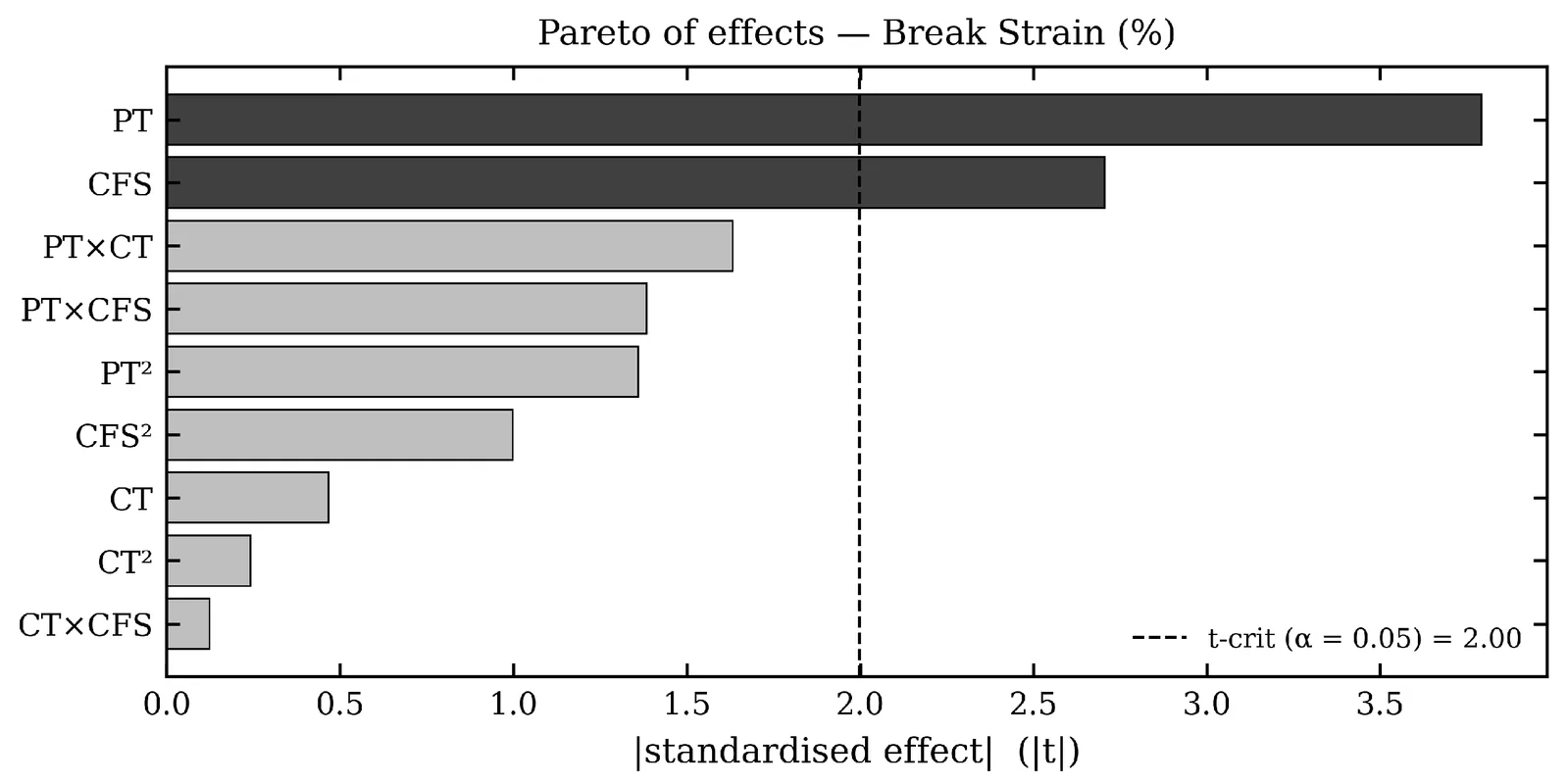
Pareto plot of standardized effects for break strain.

The relative importance of every term in the second-order model is summarized by the Pareto plots in Figure 8, Figure 9 and Figure 10. Each bar gives the absolute value of the standardized effect (|t|) of one model term; the vertical dashed line marks the t-critical value (≈2.00) at α=0.05. Bars to the right of this line correspond to statistically significant terms (dark shading); bars to the left are not significant (light shading).

The Pareto plots make the factor ranking unambiguous. PT is the single most influential term for every response. The second-ranked term differs by response: for UTS it is the CT × CFS interaction; for toughness and $\varepsilon_{B}$ it is the CFS main effect. CT does not enter as a significant main effect for any of the three responses.

### 3.3. Two-Factor Interactions

Of the three possible pairwise interactions (PT × CT, PT × CFS, CT × CFS), only the chamber-temperature × cooling-fan-speed coupling reaches statistical significance, and it does so for only one of the three responses (UTS). Table 9 summarizes the interaction F-statistics and *p*-values.

For UTS the CT × CFS interaction yields F=9.13, p=0.004 and accounts for 7.5% of the total sum of squares—the second-largest contribution after the PT main effect. For toughness this interaction is not significant (F=0.01, p=0.918). For break strain none of the pairwise interactions is statistically significant (all p>0.10). Toughness and break strain therefore both behave essentially additively in PT and CFS over the tested range, unlike UTS.

Physically, the CT × CFS interaction observed for UTS can be understood as a competition between two cooling mechanisms. At low chamber temperature (CT = 42 °C) the freshly extruded bead is already in a steep thermal gradient with respect to the surrounding chamber air; a high fan speed (CFS = 50%) further accelerates layer-by-layer quenching, reducing residual thermal stresses and promoting tight interlayer cohesion. At high chamber temperature (CT = 58 °C) the chamber is deliberately holding the part close to the polymer’s annealing range to allow gradual cooling and matrix relaxation; aggressive forced convection at CFS = 50% now competes with this controlled cooling and disrupts it, reducing strength. The interaction plot in Figure 5 shows this effect clearly as crossing lines in the CT × CFS panel; no equivalent crossing is seen for toughness (Figure 6), since its CT × CFS interaction is not statistically significant (p=0.918). The practical implication is that, for strength specifically, chamber temperature and fan speed must be optimized jointly: tuning one without the other can move UTS in unexpected directions.

### 3.4. Regression Equations

The fitted second-order regression equations expressed in real (uncoded) engineering units are given in Equations (2)–(4) below. PT and CT are in °C and CFS in %; the equations are valid within the experimental ranges of Table 6. Coefficients of terms not retained as statistically significant are nonetheless reported for completeness.

(2) $$\begin{array}{cc} \mathrm{UTS}= & -39.70+0.4646\;\cdot \;\mathrm{PT}+0.5998\;\cdot \;\mathrm{CT}+0.2136\;\cdot \;\mathrm{CFS}\\ \; & -8.466\times 10^{-4}\;\cdot \;{\mathrm{PT}}^{2}-1.050\times 10^{-3}\;\cdot \;{\mathrm{CT}}^{2}+2.725\times 10^{-4}\;\cdot \;{\mathrm{CFS}}^{2}\\ \; & -1.879\times 10^{-3}\;\cdot \;\mathrm{PT}\;\cdot \;\mathrm{CT}-3.550\times 10^{-4}\;\cdot \;\mathrm{PT}\;\cdot \;\mathrm{CFS}-2.560\times 10^{-3}\;\cdot \;\mathrm{CT}\;\cdot \;\mathrm{CFS} \end{array}$$

(3) $$\begin{array}{cc} T= & -41.94+0.3392\;\cdot \;\mathrm{PT}+0.1821\;\cdot \;\mathrm{CT}+0.0770\;\cdot \;\mathrm{CFS}\\ \; & -6.174\times 10^{-4}\;\cdot \;{\mathrm{PT}}^{2}+8.834\times 10^{-4}\;\cdot \;{\mathrm{CT}}^{2}+2.923\times 10^{-4}\;\cdot \;{\mathrm{CFS}}^{2}\\ \; & -1.105\times 10^{-3}\;\cdot \;\mathrm{PT}\;\cdot \;\mathrm{CT}-3.258\times 10^{-4}\;\cdot \;\mathrm{PT}\;\cdot \;\mathrm{CFS}-5.736\times 10^{-5}\;\cdot \;\mathrm{CT}\;\cdot \;\mathrm{CFS} \end{array}$$

(4) $$\begin{array}{cc} \varepsilon_{B}= & -164.14+1.3292\;\cdot \;\mathrm{PT}+0.7352\;\cdot \;\mathrm{CT}+0.2384\;\cdot \;\mathrm{CFS}\\ \; & -2.424\times 10^{-3}\;\cdot \;{\mathrm{PT}}^{2}+2.693\times 10^{-3}\;\cdot \;{\mathrm{CT}}^{2}+1.138\times 10^{-3}\;\cdot \;{\mathrm{CFS}}^{2}\\ \; & -4.123\times 10^{-3}\;\cdot \;\mathrm{PT}\;\cdot \;\mathrm{CT}-1.119\times 10^{-3}\;\cdot \;\mathrm{PT}\;\cdot \;\mathrm{CFS}+2.501\times 10^{-4}\;\cdot \;\mathrm{CT}\;\cdot \;\mathrm{CFS} \end{array}$$

For prediction at new, untested operating points, the reduced equations below—retaining the statistically significant terms from Table 10, together with the CT main effect for UTS to preserve model hierarchy given its significant CT × CFS interaction—are recommended in place of Equations (2)–(4). Condition-level (leave-one-condition-out) cross-validation shows that these reduced equations predict held-out conditions with roughly half the root-mean-square error of the full quadratic equations above (UTS: 1.67 → 0.85 MPa; toughness: 0.84 → 0.43 MJ/m^3^; break strain: 2.86 → 1.53%), consistent with the full equations overfitting the limited number of unique design points relative to the number of estimated coefficients. Equations (2)–(4) remain the appropriate reference for characterizing which effects are statistically significant (Section 3.5) and for the multi-response optimization of Section 3.8, both of which depend on the complete term set; the reduced equations below (Equations (5)–(7)) are intended specifically for point prediction at new PT/CT/CFS combinations.

(5) $$\mathrm{UTS}=35.04-0.04462\;\cdot \;\mathrm{PT}+0.06400\;\cdot \;\mathrm{CT}+0.1420\;\cdot \;\mathrm{CFS}-2.560\times 10^{-3}\;\cdot \;\mathrm{CT}\;\cdot \;\mathrm{CFS}$$

(6) T=6.706−0.02054·PT+0.01059·CFS

(7) $$\varepsilon_{B}=24.93-0.06854\;\cdot \;\mathrm{PT}+0.03913\;\cdot \;\mathrm{CFS}$$

### 3.5. ANOVA and Model Adequacy

Table 10 summarizes, for every response, the terms that are statistically significant at α=0.05 in the Type-II ANOVA, together with their F-statistic, *p*-value and the sign of the underlying linear (or interaction) coefficient.

Table 10 shows that CT does not appear as a statistically significant term, either as a main effect or in interaction, in the final models for toughness or $\varepsilon_{B}$, and enters the model only through the CT × CFS interaction for UTS (Table 9). By contrast, CFS has a significant positive main effect on all three responses. This pattern is consistent with the flat build orientation tested here (Section 4): crack propagation must traverse the raster and bulk material rather than the weaker interlayer interface, so responses that depend on crack propagation (toughness, $\varepsilon_{B}$) may be primarily governed by within-layer factors such as CFS, while CT’s influence, acting on the longer-time-scale interlayer thermal history, is only detected where it interacts with CFS to affect peak load (UTS). In this study, chamber temperature therefore had no statistically significant effect on toughness or break strain, either alone or in interaction with other factors, within the tested range (42–58 °C), flat orientation and n=5 replicates per condition; this should be read as an absence of a detected CT effect under these conditions, not as evidence that CT has no effect on FDM ABS in general, and should be interpreted as a consequence of the tested orientation rather than a general property of the material (see Section 4).

**Figure 11:**
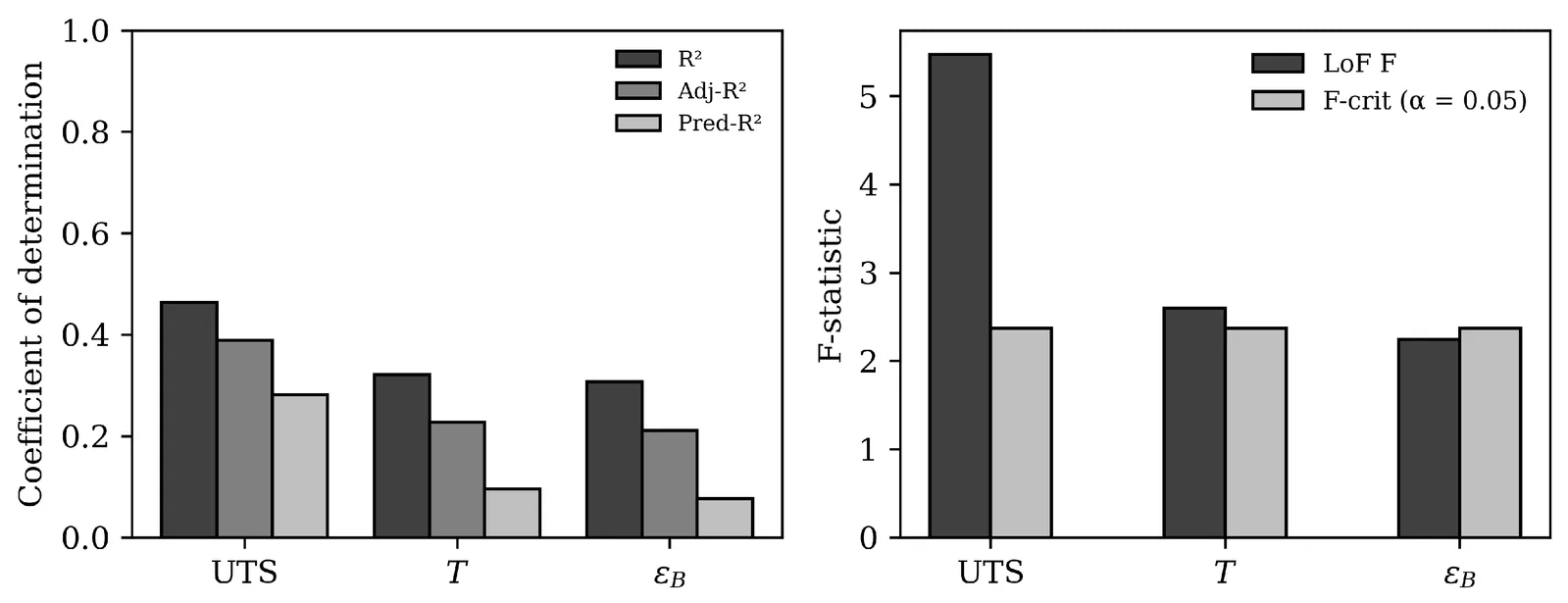
Goodness-of-fit indicators (**left**) and lack-of-fit F-statistic vs. its α=0.05 critical value (**right**) for the three models.

The global fit statistics are summarized in the left panel of Figure 11. The coefficients of determination are R^2^ = 0.46/0.32/0.31 (adjusted R^2^ = 0.39/0.23/0.21) for UTS, toughness and $\varepsilon_{B}$ respectively. These values are dominated by the inherent replicate scatter quantified in Section 3.1, which is irreducible within this experimental design. Any additional unexplained variance attributable to structural inadequacy of the quadratic surface was further assessed via the lack-of-fit F-test described in Section 2.5 (comparing MS_*LoF*_ against MS_*PE*_), with results summarized in Table 11. As a further, more conservative check of predictive capability, the leave-one-out PRESS-based predicted R^2^ is 0.28/0.10/0.08 for UTS, toughness and $\varepsilon_{B}$ respectively—lower than the adjusted R^2^ in each case, indicating that the fitted surfaces predict untested conditions less precisely than they fit the 75 observations used to build them. This gap is consistent with the pure-error dominance already identified above rather than with overfitting: variance inflation factors for every coded term remain below 1.3 across all three models, so multicollinearity among PT, CT and CFS is not a contributing cause.

The lack-of-fit test rejects the adequacy of the quadratic surface for UTS (p<0.001) and toughness (p=0.034), but not for break strain (p=0.062>0.05). The data therefore contain some systematic curvature beyond the fitted second-order polynomial in the strength and toughness responses—most plausibly along the PT axis, where the main-effect plots in Figure 5 and Figure 6 show a small but visible non-monotonicity between 220 °C and 240 °C. However, the magnitude of the lack-of-fit contribution remains modest relative to the pure error (SS_*LoF*_/SS_*PE*_ = 0.46 for UTS, 0.22 for toughness), and the quadratic surface still captures the dominant physical trends and significant interactions. The second-order model is therefore retained as a first-order engineering approximation, and higher-order or piecewise refinement of the PT response—through finer sampling between 220 °C and 240 °C—is identified as a clear target for a follow-up study.

### 3.6. Residual Diagnostics

**Figure 12:**
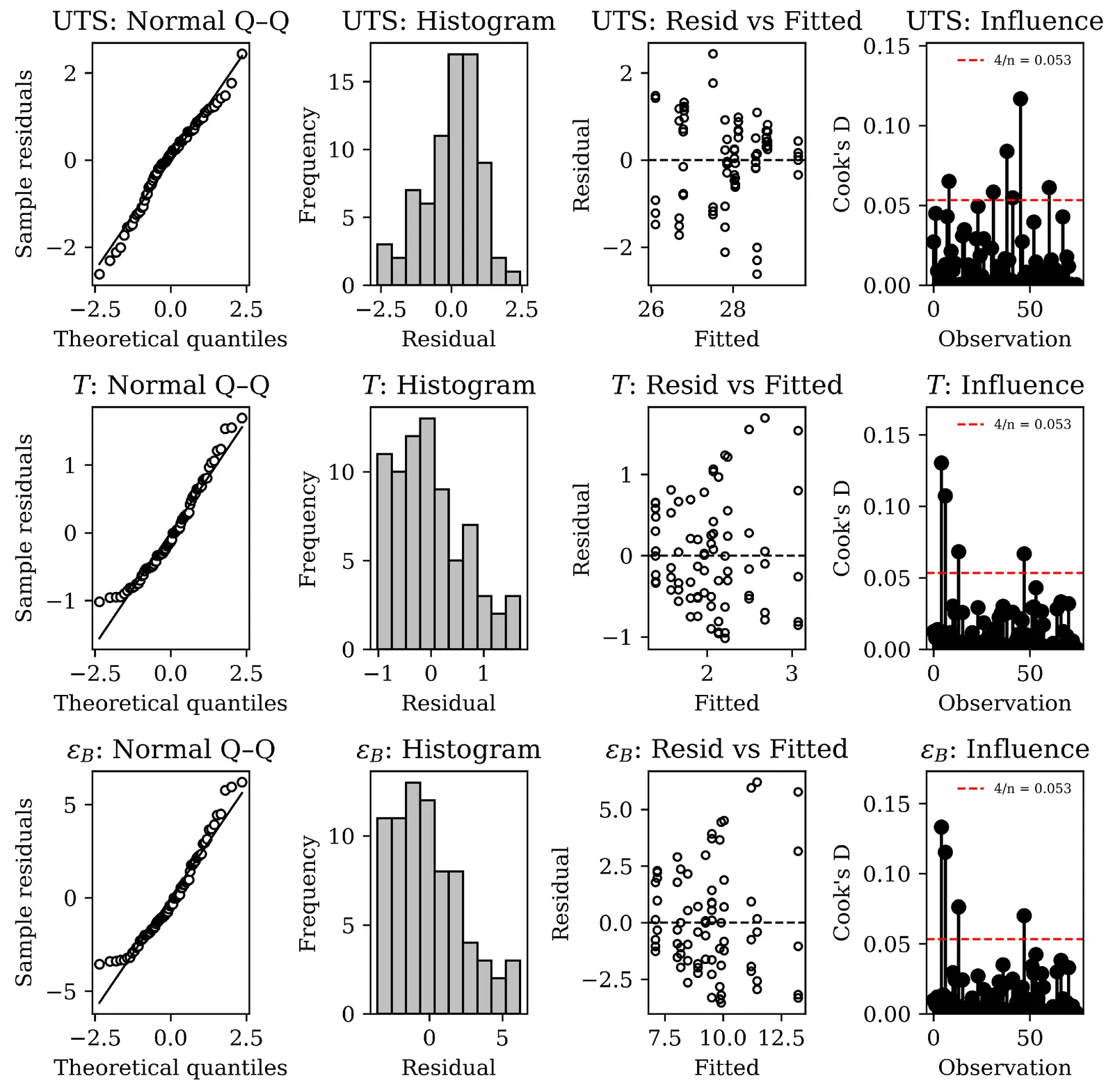
Residual diagnostics for UTS, toughness and break strain (rows): Q–Q plot, histogram, residuals vs. fitted, and Cook’s distance. Circles are individual observations (75 specimens); the solid line in the Q–Q plots is the normal reference line, the dashed line in the residuals-vs.-fitted plots marks zero residual, and the dashed line in the Cook’s distance plots marks the 4/n influence threshold (n=75).

The validity of the OLS regression rests on three assumptions about the residuals: approximate normality, constant variance across the design region, and absence of high-leverage outlying observations. Each assumption was tested directly. Table 12 lists the test outcomes; Figure 12 shows the corresponding four-in-one diagnostic plots.

The Shapiro–Wilk test does not reject normality for UTS (p=0.24), but does flag a departure for toughness (p=0.012) and for break strain (p=0.005), consistent with the right-skew visible in their residual histograms in Figure 12. Because the OLS coefficient estimates remain unbiased under non-normal errors and the sample size (n=75) is large enough for the central-limit theorem to support approximate normality of the slope estimators, the inferences remain robust to this departure. The Breusch–Pagan test confirms the equal-variance assumption for toughness and $\varepsilon_{B}$, but rejects it for UTS (p<0.001), in agreement with Levene’s test in Section 3.1; heteroscedasticity-consistent (HC3) standard errors used as a sensitivity check do not alter the set of statistically significant terms reported in Table 10. A small number of observations (3–6 per response) exceed the 4/*n* Cook’s-distance threshold, but the maximum value (D=0.13) is well below unity and the model coefficients change by less than 5% when these observations are removed. We therefore retain all 75 observations in the reported fits.

### 3.7. Effect-Size Quantification

Significance testing alone is silent on practical magnitude: a term may be statistically significant yet account for a negligible share of the response variance, especially when n=75. To distinguish significance from importance, three complementary effect-size measures were computed from the Type-II ANOVA: the percentage of the total sum of squares (%SS), the partial $\eta^{2}$ (ratio of term-SS to the sum of term-SS and residual-SS), and the bias-corrected $\omega^{2}$. The dominant terms are listed in Table 13.

**Figure 13:**
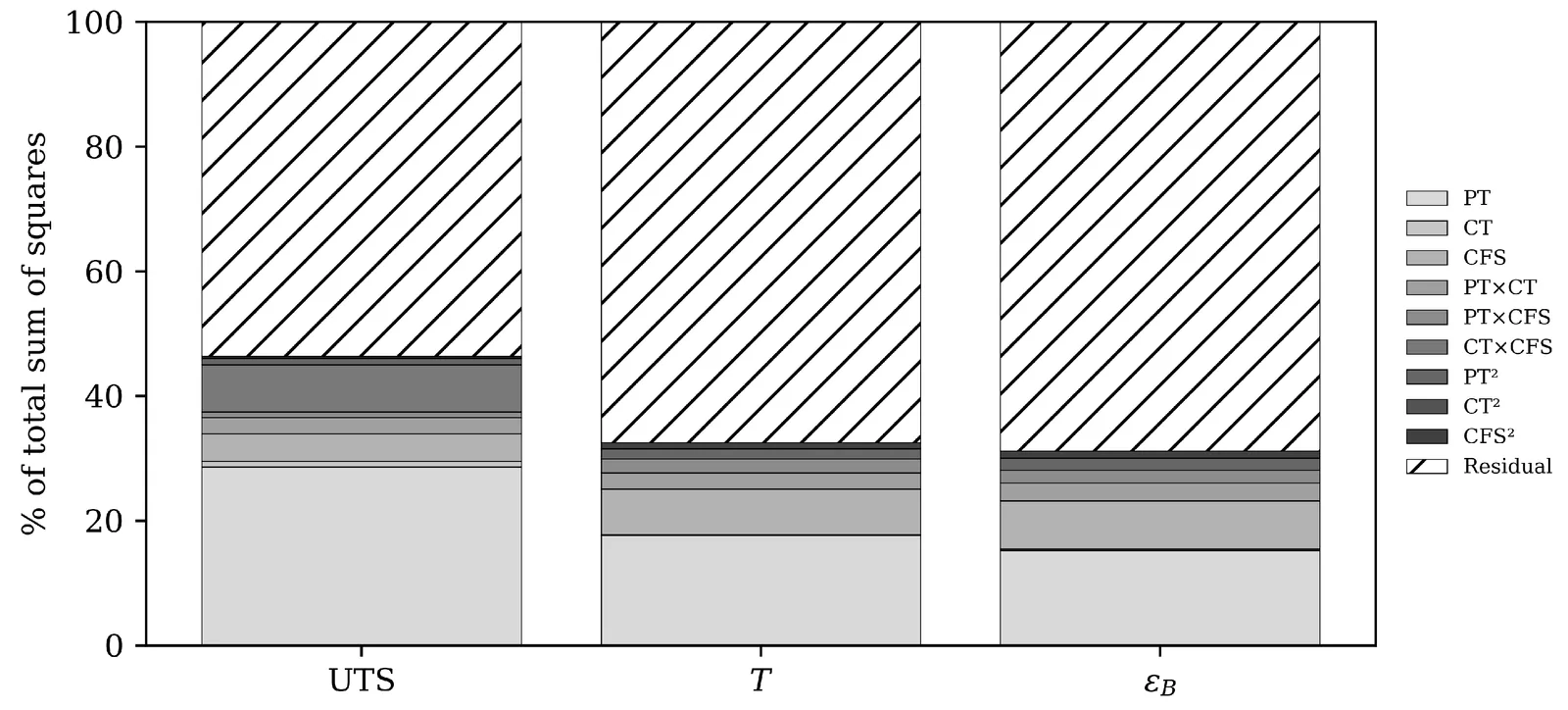
Percentage contribution of each model term to the total sum of squares of each response (UTS, *T* and $\varepsilon_{B}$); the hatched segment is the residual (unexplained) variance.

**Figure 14:**
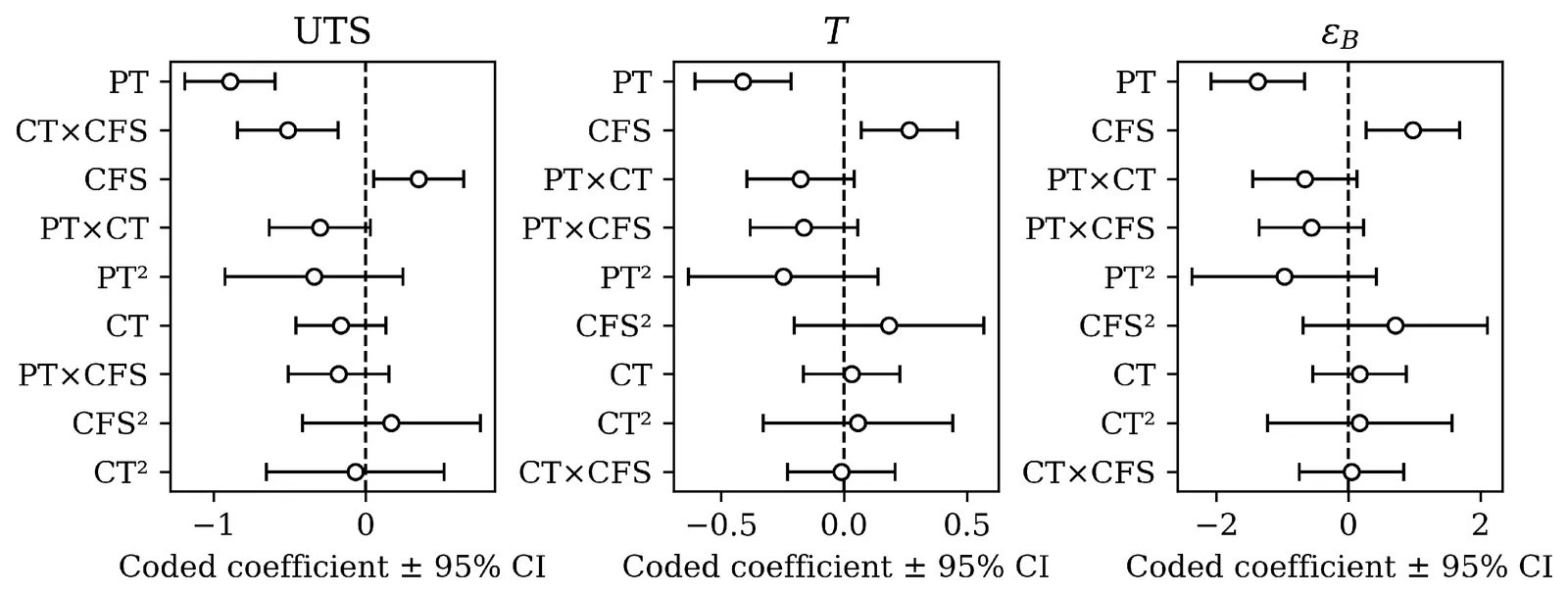
Forest plot of coded regression coefficients and 95% CIs for UTS, toughness and break strain; CIs excluding zero are significant at α=0.05. Circles are the point estimates, horizontal bars are the 95% CIs, and the vertical dashed line marks zero (no effect).

PT is by far the largest single contributor for every response, accounting for 15% to 29% of the total response variance on its own. The CT × CFS interaction is the second-most important term for UTS alone ($\eta_{p}^{2}=0.12$), reinforcing the conclusion of Section 3.3 that chamber temperature and fan speed cannot be optimized independently for strength. For both toughness ($\eta_{p}^{2}=0.10$) and break strain ($\eta_{p}^{2}=0.10$) the second-ranked term is instead the CFS main effect. Each individual $\omega^{2}$ value is modest (<0.3 throughout)—a direct consequence of the substantial replicate scatter quantified in Section 3.1—but the relative ranking is unambiguous and informs the optimization strategy of Section 3.8. Figure 13 visualizes the full %SS breakdown for every model term, including those not individually significant, and Figure 14 presents the same coded coefficients as a forest plot with 95% confidence intervals.

### 3.8. Single- and Multi-Response Optimization

Maximizing each fitted response surface in isolation yields the individual optima of Table 14. UTS is maximized by a low-temperature/high-cooling recipe (PT = 220 °C, CT = 42 °C, CFS = 50%), while both toughness and break strain instead prefer a hot chamber (CT = 58 °C) with the same PT and CFS settings.

A practical printing recipe must reconcile the three competing requirements. The Derringer–Suich desirability approach was therefore applied: every predicted response $Y_{i}$ is mapped to an individual desirability $d_{i}\in \left[0,1\right]$ using the “larger-is-better” linear transform anchored at the observed minimum and maximum of the experimental data, and the overall desirability *D* is the weighted geometric mean of the $d_{i}$. *D* was maximized over the coded factor cube by a multi-start L-BFGS-B search. The full quadratic model (Equations (2)–(4)) was used because it estimates all quadratic and interaction terms directly, but Table 11 shows a statistically significant lack of fit for UTS and toughness, so the optimization was repeated with the reduced, statistically-significant-terms-only equations (Equations (5)–(7)), which cross-validate better on held-out conditions (Section 3.4). Both are reported in Table 15, together with a weight sweep that increases the relative weight on UTS to test the robustness of the composite optimum.

Under the full model with equal weighting, the global optimum is at PT = 220 °C, CT = 58 °C, CFS = 50%, with overall desirability D≈0.63, since both toughness and break strain have their (non-significant) point estimates at the hot-chamber setting. This optimum is unchanged up to a UTS weight of 2; at a UTS weight of 3 the optimum moves to an intermediate CT = 53 °C, and at a UTS weight of 4 or higher it moves to CT = 42 °C, matching the UTS-only optimum. The composite optimum therefore only flips once UTS is weighted roughly three times as heavily as each of the other two responses individually; earlier scenarios in Table 15 (maximum UTS weight of 2) did not reach this ratio, which is why the optimum did not move for them. In the reduced model, by contrast, CT = 42 °C is the optimum at every weighting tested, because the reduced equations do not retain a CT term for toughness or break strain. In every scenario and every model, PT and CFS remain pinned at their experimental extremes (220 °C and 50%), so these two recommendations are robust to both the choice of weighting and the choice of model; only the recommended CT setting is sensitive to these choices.

As a partial confirmation check, the full-model hot-chamber recipe (PT = 220 °C, CT = 58 °C, CFS = 50%) is not a model extrapolation: it corresponds exactly to run S07, one of the fifteen physically tested conditions, with five replicates already collected. The as-measured means at S07 are UTS = 29.3 MPa, toughness = 3.16 MJ/m^3^ and $\varepsilon_{B}$ = 13.5%, compared with full-model predictions of 28.8 MPa, 3.07 MJ/m^3^ and 13.2% respectively—differences of 0.5 MPa, 0.09 MJ/m^3^ and 0.3 percentage points, all within the pooled repeatability SD reported in Table 8. However, agreement between the model prediction and the S07 measurement at a single condition does not, by itself, establish that the difference between CT settings is statistically resolvable, which requires a direct contrast.

A formal contrast between CT = 58 °C and CT = 42 °C (at PT = 220 °C, CFS = 50%) was therefore computed with 95% confidence intervals, both from the full model’s predicted means and from the measured means of the corresponding tested conditions, S07 (CT = 58 °C) and S05 (CT = 42 °C) (Table 16).

All four intervals include zero, so the predicted gain of roughly 15% in toughness and break strain from raising CT to 58 °C cannot be separated from noise at this replication level, consistent with the absence of a significant CT term for these two responses in Table 10 and with the power analysis of Section 3.1. CT is accordingly treated as unresolved for toughness and break strain within this design, and the hot-chamber setting is reported only as the (statistically indistinguishable) point estimate from the full model, not as an established improvement.

## 4. Discussion

Three findings warrant explicit discussion. First, PT dominated all three responses, and its effect was negative on every response within the tested range. The mechanism behind this trend was not determined in this study. Possible explanations include thermally induced changes in the material and changes in melt viscosity, bead geometry and cooling behavior, but none of these was characterized, and they would need to be tested with, for example, thermal analysis (TGA, DSC), spectroscopy (FTIR), rheology or melt-flow measurements, and microstructural analysis. Because material degradation, porosity and interlayer bond quality were not characterized in this study, these mechanisms should be regarded as plausible interpretations rather than established causes. Within the tested range, the results suggest that process design should favor the lower end of the manufacturer’s recommended extrusion window (220 °C) rather than the mid-to-high range. However, the modest non-monotonicity detected near 220–240 °C (Section 3.5) and the absence of data below 220 °C mean this recommendation should not be extrapolated toward lower, untested temperatures without further verification. Excessively low extrusion temperatures are also reported to impair interlayer bonding by increasing melt viscosity and limiting wetting and molecular diffusion, a competing effect this study did not characterize.

Second, the CT × CFS interaction has both engineering and physical significance. The absence of a significant PT × CT or PT × CFS interaction at the resolution of the present design suggests that PT can be optimized largely independently of the other two factors, a useful simplification for industrial process control. The CT × CFS coupling may reflect the fact that the two parameters control overlapping aspects of the local thermal history: CT sets the long-time-scale ambient temperature around the part, while CFS governs short-time-scale heat removal at the layer interface. A plausible interpretation is that in a cold chamber, more aggressive convective cooling reinforces the natural quench, whereas in a hot chamber the same fan disrupts the intended slow-cool environment. This interpretation was not verified by direct measurement of the interlayer temperature or bond quality. Competitive couplings of this kind are what response-surface designs with axial points are well suited to detect and quantify.

It should be noted that this interaction was observed under flat-orientation loading (Table 5), where the tensile load is carried mainly by the raster-to-raster bonds and the polymer matrix in the plane of the layers rather than by the inter-layer (Z) bond. All specimens were printed in the flat orientation (XY plane) with the tensile load applied along the X-axis. The four perimeter shells therefore ran parallel to the load, while the infill rasters, which made up approximately three quarters of the gauge cross-section, were oriented at $\pm 45^{\circ }$ to it, so load transfer depended mainly on raster-to-raster bonding, and the inter-layer (Z) bond was not loaded in direct tension. Because CT and CFS act directly on the thermal history between successive layers, this is a specific limitation with respect to the loading condition these parameters would be expected to influence most: it is plausible that testing in the vertical (Z) orientation, where failure is dominated by inter-ply delamination, would show CT, CFS and their interaction to be significant across all three responses, rather than only through the interaction reported here. This was not tested in the present study. Only one raster orientation and angle were tested, which further limits the generality of the conclusions with respect to loading dominated by inter-layer bonding. A companion study in the vertical orientation is recommended to test this directly.

This distinction is consistent with fracture mechanics considerations. In the flat orientation, a propagating crack must traverse the raster and bulk material, so the measured toughness and break strain reflect the combined resistance of the material and the raster-to-raster bonds. In the vertical orientation, failure is reported to occur predominantly by delamination along the interlaminar interface, which offers a lower-energy crack path than the bulk material [17]. Because CT and CFS act directly on the quality of this interlaminar interface, their effect on crack propagation energy, and hence on toughness and break strain, would be expected to be more pronounced and more consistent in the vertical orientation than observed here. Table 10 shows that CT does not appear as a significant term, either as a main effect or in interaction, in the final models for toughness or $\varepsilon_{B}$, and enters the model only through the CT × CFS interaction for UTS (Table 9). By contrast, CFS has a significant positive main effect on all three responses. This pattern is consistent with the flat orientation tested here: crack propagation must traverse the raster and bulk material rather than the weaker interlayer interface, so responses depending on crack propagation (toughness, $\varepsilon_{B}$) may be primarily governed by within-layer factors such as CFS, while CT’s influence, acting on longer-time-scale interlayer thermal history, is only detected where it interacts with CFS to affect peak load (UTS). The absence of a CT effect on toughness and $\varepsilon_{B}$ observed here should therefore be interpreted as a consequence of the tested orientation rather than a general property of FDM ABS.

Third, the chamber temperature range (42–58 °C) was determined by the physical limit of the printer and not by a criterion related to the material; it is well below the glass transition temperature of ABS (≈100–105 °C). The cooling fan speed was tested at 0, 25 and 50%, that is, only over the lower half of the possible 0–100% range, because ABS is normally printed with little or no part cooling. The reported models and optima for UTS, break strain and toughness therefore apply only within the tested region of the design space and should not be extrapolated to chamber temperatures closer to Tg or to fan speeds above 50%, where the behavior may differ. The highest chamber temperature tested (58 °C) was also close to the rated limit of the printer (60 °C). Temperature was verified with a thermocouple placed near the extruder and the chamber was preheated for over 30 min before each print, but it was not continuously logged, and its spatial distribution was not mapped, so small fluctuations during printing and gradients relative to the specimen location cannot be excluded. Future work should include continuous thermal monitoring and temperature mapping, particularly near the equipment’s upper limit.

Fourth, the contrast in repeatability across the three responses carries an important methodological message: UTS is highly repeatable (CV ≈ 3.3%), whereas both break strain (CV ≈ 26%) and toughness (CV ≈ 33%) are markedly noisier. Both ductility- and fracture-energy-based measurements in FDM will always be noisier channels than peak-load-based measurements such as UTS, because they depend on the full post-yield trajectory and its sensitivity to local defects, so reported $\varepsilon_{B}$ and toughness optima must be interpreted with their confidence intervals firmly in view. In the present study the 95% CI on the $\varepsilon_{B}$ optimum spans 11.2 to 15.3%, i.e., approximately ±16% of the point estimate, and the 95% CI on the toughness optimum spans 2.51 to 3.64 MJ/m^3^, i.e., approximately ±18% of the point estimate—both wide but still informative. The sensitivity power analysis of Section 3.1 and the formal CT contrast of Section 3.8 show that this scatter, rather than a genuine absence of a CT effect on toughness and break strain, is the most likely reason that a hot-chamber advantage could not be resolved for these two responses at n=5.

Three methodological limitations should also be acknowledged. (i) The lack-of-fit test detected statistically significant residual curvature in the UTS and toughness surfaces (Section 3.5), most plausibly along the PT axis. The fitted second-order model is therefore an engineering first approximation; a follow-up campaign sampling PT at four or five levels between 215 °C and 245 °C would resolve any cubic structure. (ii) All recommended optima lie on the boundary of the explored design region, meaning further improvement is plausible by extending the experimental window—provided extrudability, dimensional fidelity and printer safety limits permit. (iii) Porosity, interlayer bond quality, surface roughness, warpage and dimensional accuracy were not measured in this study. Impact strength was also not measured, and no conclusions about impact behavior are drawn; the toughness reported here is the tensile energy absorbed per unit volume up to fracture. Fracture surfaces were not examined by optical or scanning electron microscopy; such examination could have provided direct information on fracture morphology, interlayer separation and local defects, though it would not address polymer crystallinity, which is not a primary structural parameter for ABS given its predominantly amorphous character. The thermal and chemical state of the material (e.g., via DSC, TGA or FTIR) was likewise not characterized. As a result, the interpretations relating the observed tensile behavior to thermal history and interlayer bonding are based on the mechanical response and on precedent in the literature, rather than on direct structural or chemical evidence, and should be verified in future work through the combined use of these techniques.

## 5. Conclusions

Print temperature (PT) is the dominant process parameter for all three mechanical responses, accounting for 15–29% of the total response variance, with a consistently negative effect: lower PT yields higher strength, toughness and ductility.

The CT × CFS interaction is statistically significant for UTS (p=0.004) but not for toughness (p=0.918) or break strain (p=0.902).

In this study, chamber temperature had no statistically significant effect on toughness or break strain, either as a main effect or in interaction, within the tested range (42–58 °C), flat orientation and n=5 replicates per condition.

Chamber temperature and cooling-fan speed must therefore be optimized jointly for strength.

CT’s effect on toughness and ductility could not be resolved from noise at this replication level; this should not be read as evidence that CT has no effect on FDM ABS in general, and is plausibly a consequence of the flat orientation tested here rather than a general property of the material.

PT is statistically independent of the other two factors at the resolution of this design.

The process is highly repeatable for strength (CV ≈ 3.3%) but intrinsically more variable for toughness and ductility (CV ≈ 33% and ≈26% respectively), reflecting the local-defect-driven nature of both fracture energy and elongation in FDM parts. A sensitivity power analysis confirms that five replicates per condition are adequate to resolve the effects reported for UTS but under-powered for smaller effects on toughness and break strain. UTS data show mild heteroscedasticity but the headline conclusions are robust under heteroscedasticity-consistent standard errors.

PT = 220 °C and CFS = 50% are robust recommendations, unchanged across the full and reduced models and across all desirability weightings tested. The optimal chamber temperature, by contrast, is not resolved for toughness and break strain: a formal contrast between CT = 58 °C and CT = 42 °C (Table 16) gave 95% confidence intervals that included zero for both responses, so the point-estimate gain from the hot-chamber setting cannot be distinguished from noise at this replication level. For UTS the cold-chamber setting (CT = 42 °C) is a well-resolved optimum (29.6 MPa, versus 28.8 MPa at CT = 58 °C). If maximum strength is the design priority, or if no CT-specific evidence for toughness or break strain is available, PT = 220 °C, CT = 42 °C, CFS = 50% is therefore the recommended, statistically resolved recipe; the hot-chamber setting (CT = 58 °C) remains a reasonable choice only if the (unresolved) point estimates for toughness and break strain are weighted heavily relative to strength, and should be understood as such rather than as an established improvement.

All recommended optima lie on the boundary of the explored design region, indicating that further mechanical improvement may be accessible by extending the experimental window to PT < 220 °C and CFS > 50%, subject to extrudability and dimensional fidelity constraints. A confirmation run at the recommended setting with at least five replicates is recommended before transfer to production. Additional replicates and a companion study in the vertical (Z) orientation are recommended to resolve the CT effect on toughness and break strain more definitively.

## Figures and Tables

**Table 1:** Material properties of eSUN ABS filament (eSUN TDS V4.0, 2021) [14].

Category	Property	Value	Unit
Physical	Density	1.04	g/cm^3^
Physical	Melt Flow Rate (190 °C/2.16 kg)	12	g/10 min
Mechanical	Tensile Strength	40	MPa
Mechanical	Elongation at Break	22	%
Thermal	Heat Distortion Temp. (0.455 MPa)	78	°C

**Table 2:** Manufacturer-recommended printing conditions for eSUN ABS 1.75 mm filament.

Parameter	Recommended Range/Value
Nozzle (Extrusion) Temperature	220–260 °C
Build Platform (Bed) Temperature	90–110 °C

**Table 3:** Nominal dimensions of the ASTM D638 Type I tensile specimen.

Dimension	Value (mm)
Width of Narrow Section (W)	13
Length of Narrow Section (L)	57
Width Overall (WO)	19
Overall Length (LO)	165
Gauge Length (G)	50
Distance Between Grips (D)	115
Radius of Fillet (R)	76
Thickness (T)	4.8

**Table 4:** Technical specifications of the QIDI i-Fast FDM 3D printer.

Specification	Detail
Manufacturer	QIDI Technology Co., Ltd.
Model	i-Fast
Print Technology	FDM (Fused Deposition Modeling)
Build Volume (X × Y × Z)	330 × 250 × 320 mm
Nozzle Diameter	0.4 mm (standard brass)
Filament Diameter	1.75 mm
Max. Nozzle Temperature	300 °C
Max. Bed Temperature	100 °C
Max. Chamber Temperature	60 °C (actively heated)
Layer Thickness Range	0.05–0.40 mm
Printing Speed Range	30–150 mm/s
Frame/Enclosure	Fully enclosed, metal frame
Motion System	Dual Z-axis, all-linear guide rails
Slicing Software	QIDI Print (Cura-based)
Connectivity	Wi-Fi/LAN/USB

**Table 5:** Fixed printing parameters maintained constant across all experimental conditions.

Specification	Detail
Layer Height	0.2 mm
Infill Density	100%
Infill Pattern	Rectilinear (Zigzag, ±45°)
Print Speed	40 mm/s
Build Platform (Bed) Temperature	100 °C
Nozzle Diameter	0.4 mm
Number of Perimeter Shells	4
Number of Top/Bottom Layers	7
Build Orientation	Flat (XY plane, load along X-axis)
Support Structures	None

**Table 6:** Factor levels and coded values for the FC-CCD. PT = printing temperature, CT = chamber temperature, CFS = cooling fan speed.

Level	PT (°C)	CT (°C)	CFS (%)
Low (−1)	220	42	0
Center (0)	240	50	25
High (+1)	260	58	50

**Table 7:** FC-CCD experimental run matrix.

Run	Point Type	PT (°C)	CT (°C)	CFS (%)
S01	Factorial	220	42	0
S02	Factorial	260	42	0
S03	Factorial	220	58	0
S04	Factorial	260	58	0
S05	Factorial	220	42	50
S06	Factorial	260	42	50
S07	Factorial	220	58	50
S08	Factorial	260	58	50
S09	Axial	220	50	25
S10	Axial	260	50	25
S11	Axial	240	42	25
S12	Axial	240	58	25
S13	Axial	240	50	0
S14	Axial	240	50	50
S15	Center	240	50	25

**Table 8:** Repeatability and variance-partition statistics per response ($\sigma_{between}^{2}$ = share of total variance from between-condition effects; * = unequal variance at α=0.05).

Response	$\bar{Y}$	$\sigma_{\mathrm{pooled}}$	CV (%)	$\sigma_{\mathrm{between}}^{2}$ (%)	Levene *p*
UTS (MPa)	27.88	0.924	3.3	59	0.033 *
Toughness (MJ/m^3^)	2.04	0.665	32.6	47	0.841
Break Strain (%)	9.46	2.44	25.8	38	0.764

**Table 9:** Pairwise interaction statistics from the Type-II ANOVA for all three responses (* = significant at α=0.05).

Interaction	UTS		T		$\varepsilon_{B}$	
	F	*p *	F	*p *	F	*p *
PT × CT	3.15	0.081	2.52	0.118	2.66	0.108
PT × CFS	1.10	0.299	2.14	0.149	1.92	0.171
CT × CFS	9.13	0.004 *	0.01	0.918	0.02	0.902

**Table 10:** ANOVA-significant terms (α=0.05) per response.

Response	Term	F	*p*	Interpretation
UTS (MPa)	PT	34.69	<0.001	Strong negative linear effect
UTS (MPa)	CT × CFS	9.13	0.004	Significant two-factor coupling
UTS (MPa)	CFS	5.34	0.024	Mild positive linear effect
Toughness (MJ/m^3^)	PT	16.98	<0.001	Strong negative linear effect
Toughness (MJ/m^3^)	CFS	7.05	0.010	Positive linear effect
$\varepsilon_{B}$ (%)	PT	14.38	<0.001	Strong negative linear effect
$\varepsilon_{B}$ (%)	CFS	7.32	0.009	Positive linear effect

**Table 11:** Partitioned sums of squares and lack-of-fit F-test.

Response	SS_PE_	SS_LoF_	F_LoF_	*p*-Value	Conclusion
UTS (MPa)	51.25	23.37	5.47	<0.001	Significant LoF
Toughness (MJ/m^3^)	26.54	5.75	2.60	0.034	Significant LoF
$\varepsilon_{B}$ (%)	357.95	66.85	2.24	0.062	Adequate

**Table 12:** Residual diagnostic tests (n=75; n_out = observations above the 4/*n* Cook’s-distance threshold of 0.053; * = p<0.05).

Response	Shapiro *W*	Shapiro *p*	Breusch–Pagan *p*	n_out	Max *D*
UTS (MPa)	0.979	0.240	<0.001 *	6	0.117
Toughness (MJ/m^3^)	0.957	0.012 *	0.128	4	0.130
$\varepsilon_{B}$ (%)	0.950	0.005 *	0.094	4	0.133

**Table 13:** Effect-size statistics for the leading significant terms per response ($\eta_{p}^{2}$ = partial $\eta^{2}$; $\omega^{2}$ = bias-corrected $\omega^{2}$).

Response	Term	%SS	F	*p*	$\eta_{p}^{2}$	$\omega^{2}$
UTS	PT	28.6	34.69	<0.001	0.348	0.276
UTS	CT × CFS	7.5	9.13	0.004	0.123	0.067
UTS	CFS	4.4	5.34	0.024	0.076	0.036
T	PT	17.6	16.98	<0.001	0.207	0.164
T	CFS	7.3	7.05	0.010	0.098	0.062
$\varepsilon_{B}$	PT	15.2	14.38	<0.001	0.181	0.140
$\varepsilon_{B}$	CFS	7.8	7.32	0.009	0.101	0.066

**Table 14:** Single-response optima with 95% confidence intervals on the predicted mean response.

Response	PT (°C)	CT (°C)	CFS (%)	Predicted	95% CI on Mean
UTS (MPa)	220	42	50	29.6	[28.7, 30.5]
Toughness (MJ/m^3^)	220	58	50	3.07	[2.51, 3.64]
Break Strain (%)	220	58	50	13.2	[11.2, 15.3]

**Table 15:** Multi-response (desirability) optima under three weighting scenarios (full model). Also: recommended CT from a UTS-weight sweep and from the reduced model ($w_{T}=w_{\varepsilon B}=1$ throughout).

Scenario $(w_{\mathrm{UTS}}$:$w_{T}$:$w_{\varepsilon B})$	Model	PT (°C)	CT (°C)	CFS (%)	UTS	T$/\varepsilon_{B}$
Equal (1:1:1)	Full	220	58	50	28.8	3.07/13.2
Strength + T (2:2:1)	Full	220	58	50	28.8	3.07/13.2
Ductility (1:1:3)	Full	220	58	50	28.8	3.07/13.2
Weight-sweep on $w_{UTS}$ (Full model), recommended CT only:						
$w_{UTS}\leq 2$	Full	220	58	50	(as above)	
$w_{UTS}=3$	Full	220	53	50	intermediate CT	
$w_{UTS}\geq 4$	Full	220	42	50	(=UTS optimum)	
Any weighting	Reduced	220	42	50	(=UTS optimum)	

**Table 16:** Formal contrast between CT = 58 °C and CT = 42 °C at PT = 220 °C, CFS = 50%, with 95% confidence intervals.

Response	Difference (Model)	Difference (Measured, S07 − S05)
Toughness (MJ/m^3^)	+0.39 (95% CI −0.35 to 1.14)	+0.44 (95% CI −1.05 to 1.94)
Break strain (pp)	+1.76 (95% CI −0.94 to 4.46)	+1.94 (95% CI −3.70 to 7.59)

## Data Availability

The original contributions presented in this study are included in the article. Further inquiries can be directed to the corresponding author.
